# Aerobic addition of secondary phosphine oxides to vinyl sulfides: a shortcut to 1-hydroxy-2-(organosulfanyl)ethyl(diorganyl)phosphine oxides

**DOI:** 10.3762/bjoc.11.214

**Published:** 2015-10-23

**Authors:** Svetlana F Malysheva, Alexander V Artem’ev, Nina K Gusarova, Nataliya A Belogorlova, Alexander I Albanov, C W Liu, Boris A Trofimov

**Affiliations:** 1 A. E. Favorsky Irkutsk Institute of Chemistry, Siberian Branch, Russian Academy of Sciences, 1 Favorsky Str., 664033 Irkutsk, Russian Federation; 2 Department of Chemistry, National Dong Hwa University, Hualien 97401, Taiwan

**Keywords:** addition, green method, phosphine oxides, regioselectivity, vinyl sulfides

## Abstract

Secondary phosphine oxides react with vinyl sulfides (both alkyl- and aryl-substituted sulfides) under aerobic and solvent-free conditions (80 °C, air, 7–30 h) to afford 1-hydroxy-2-(organosulfanyl)ethyl(diorganyl)phosphine oxides in 70–93% yields.

## Findings

Tertiary phosphines and phosphine chalcogenides are important organophosphorus compounds that are widely used in industry, organic synthesis, polymer science, medicinal and coordination chemistry [1–4]. Therefore, the synthesis of these compounds has attracted a great interest and numerous synthetic methods have been developed [5–7]. Among them, the addition of P(X)–H (X = none, O, S or Se) to diverse alkenes is one of the most powerful and 100% atom-economic approaches to construct new C–P bonds, that provide straightforward access to tertiary phosphines and their chalcogenides [8–12]. Conventionally, the activation of the P–H bonds in this reaction is achieved by using radical initiators [13–15], Brønsted/Lewis acids [16–17] and bases [18–20] as well as transition metal catalysts [21–23]. Also, examples of the microwave-assisted [24–25] and photoinduced [26] addition are described.

**Scheme 1:**
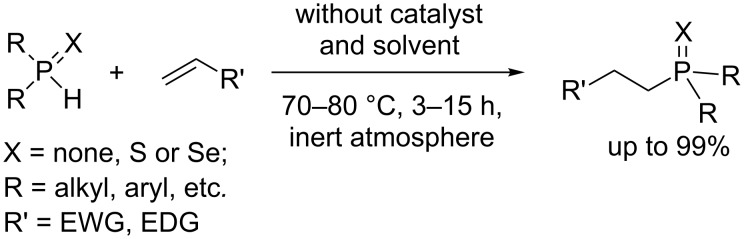
Non-catalyzed addition of P–H species to alkenes.

Recently, on example of secondary phosphines [27] as well as secondary phosphine sulfides [28] and selenides [29], it has been disclosed that the addition of P–H species to the C=C bonds readily proceeds in the absence of any catalyst or initiator (Scheme 1). The reactions occur under mild solvent-free conditions (70–80 °C, inert atmosphere, 3–15 h) to chemo- and regioselectively furnish the anti-Markovnikov adducts in excellent yields (up to 99%). The substrate scope includes both EDG- and EWG-substituted alkenes [27–29].

In this letter, we report our serendipitous finding that secondary phosphine oxides **1a–f** under aerobic conditions (air, 80 °C, 7–18 h) easily add to vinyl sulfides **2a–c** to give unknown 1-hydroxy-2-(organosulfanyl)ethyl(diorganyl)phosphine oxides **3a–h** in high yields (Table 1). The 10% excess of **2a–c** relative to **1a–f** is found to be optimal since the equimolar ratio of the reactants leads to incomplete conversion of the secondary phosphine oxides.

**Table 1:** The substrate scope for the aerobic addition of phosphine oxides **1a–f** to vinyl sulfides **2a–c**.^a^

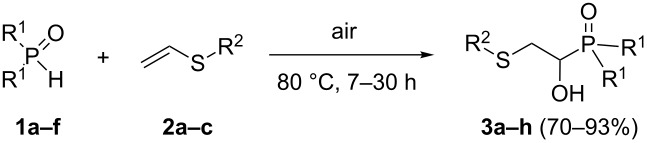				

Entry	Phosphine oxide	Vinyl sulfide	Time, h	Phosphine oxide **3a–h** (yield, %)^b^

1	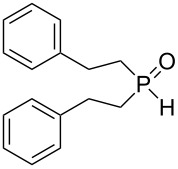 **1a**	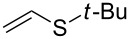 **2a**	16	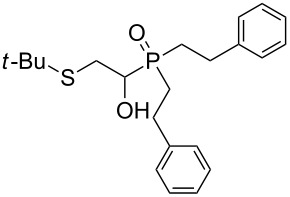 **3a** (80%)
2	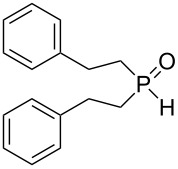 **1a**	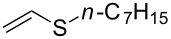 **2b**	30	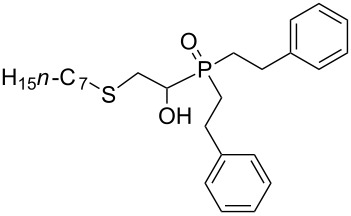 **3b** (78%)
3	Ph_2_P(O)H **1b**	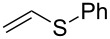 **2c**	7	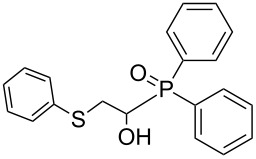 **3c** (70%)
4	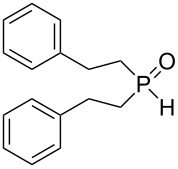 **1a**	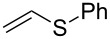 **2c**	11	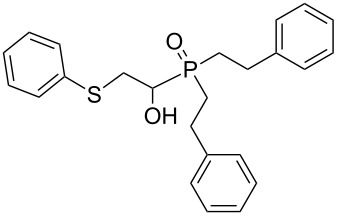 **3d** (91%)
5	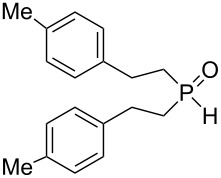 **1c**	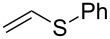 **2c**	15	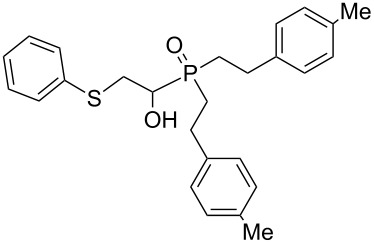 **3e** (93%)
6	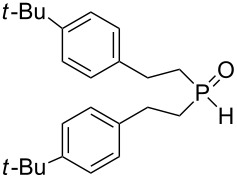 **1d**	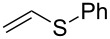 **2c**	15	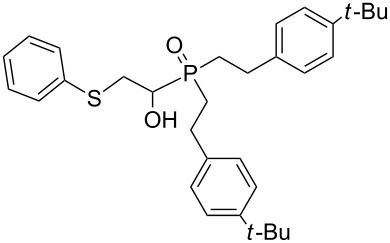 **3f** (90%)
7	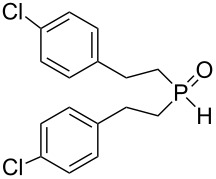 **1e**	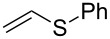 **2c**	15	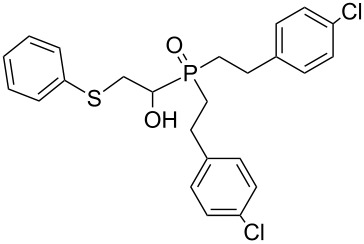 **3g** (82%)
8	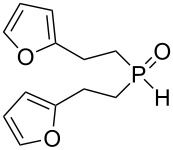 **1f**	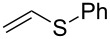 **2c**	18	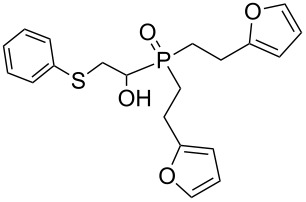 **3h** (89%)

^a^Reaction conditions: secondary phosphine oxide **1a–f** (1.0 mmol), vinyl sulfide **2a–c** (1.1 mmol) at 80 °C for 7–30 h under air. ^b^Isolated yield based on **1a–f**.

**Figure 1:**
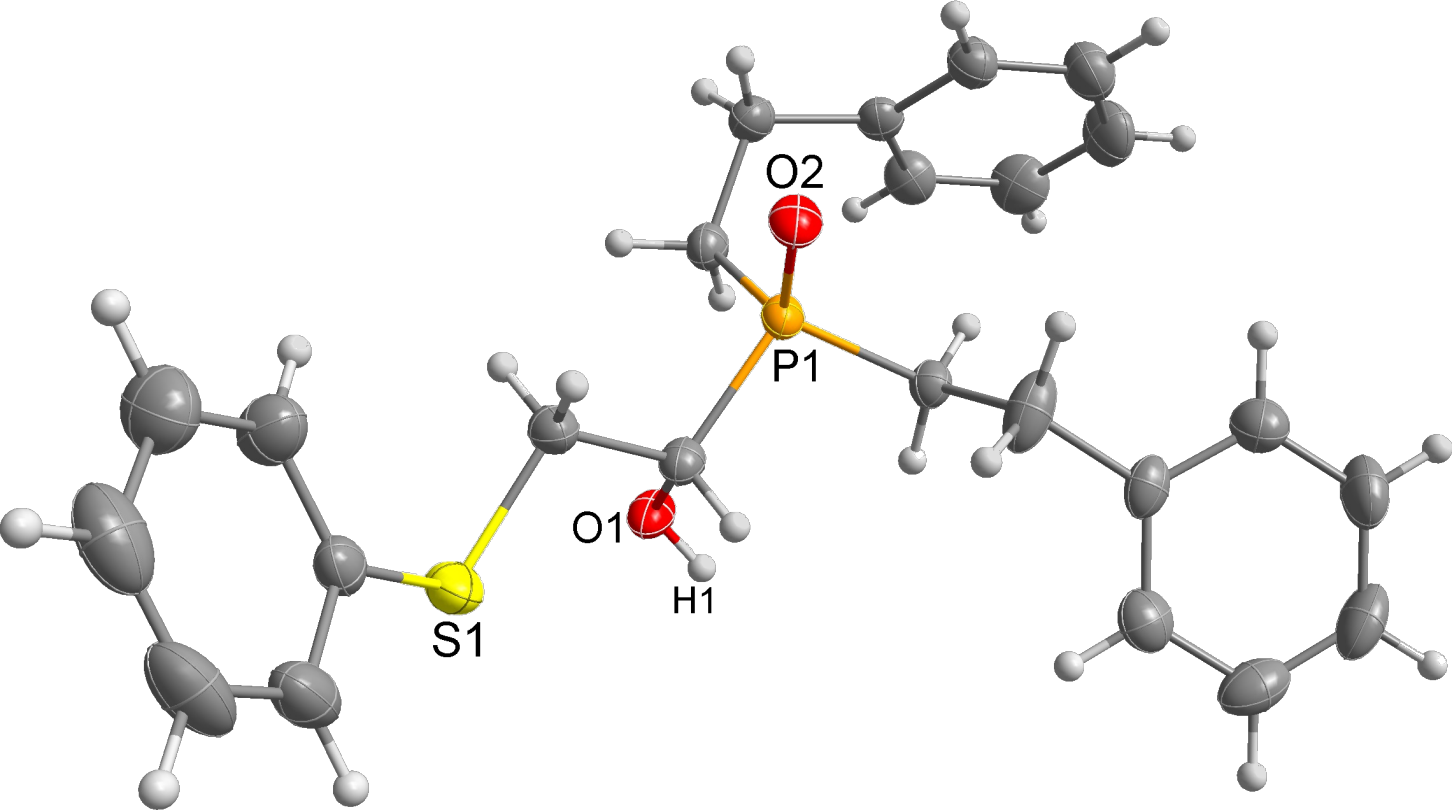
ORTEP drawing (30% thermal ellipsoid) of phosphine oxide **3d**. A CIF file with the crystallographic data is available as Supporting Information File 1 and is also available on request from the Cambridge Crystallographic Data Centre as deposition 1046604.

Importantly, under these conditions, the expected [30] anti-Markovnikov adducts are not observed in detectable amounts (^31^P NMR). The main byproducts are phosphinic acids, R_2_P(O)OH, formed by air oxidation of secondary phosphine oxides **1a–f**. As seen from Table 1, the reaction is applicable to both aryl- (**1b**) and arylalkyl-substituted (**1a**,**c–e**) secondary phosphine oxides. The furyl-containing phosphine oxide **1f** can also be reacted under these reaction conditions. On the other hand, vinyl sulfides bearing alkyl (**2a,b**) and aryl (**2c**) substituents successfully participate in the reaction to provide the corresponding phosphine oxides **3a–h**. The latter were isolated as air- and moisture-stable powders (**3a–f**) or oils (**3g**,**h**), soluble in common organic solvents. Their structures have been established by X-ray diffraction (for **3d**, Figure 1), NMR (^1^H, ^13^C, ^31^P, ^1^H,^13^C-HSQC) and FTIR techniques.

The presence of an asymmetric carbon atom in the reaction products leads to non-equivalence of both heminal protons in the SCH_2_C* fragment and carbon signals in the arylethyl moiety. In the ^1^H NMR spectra of **3a–h**, protons of the PCHCH_2_S moiety form an ABMX spin system appearing as three multiplets.

Phosphine oxide **3d** crystallizes in the centrosymmetric *P*2_1_/*c* space group. Within its extended structure, strong intermolecular H-bonding interactions between the O–H hydrogen and P=O oxygen atom of a second molecule {O(1)–H(1)···O(2), 1.80(6) Å; O–H···O angle, 174.9(7)°} leads to the formation of 1D polymeric chains along the *b*-axis (Figure S1, Supporting Information File 2).

In FTIR spectra of **3a–h**, absorption bands of the P=O and O–H bonds appear in the regions of 1100–1150 and 3350–3450 cm^−1^, respectively.

**Scheme 2:**
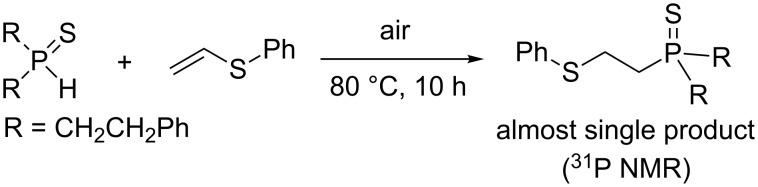
Addition of secondary phosphine sulfide to vinyl sulfide under aerobic catalyst-free conditions.

Interestingly, the reaction disclosed is specific for secondary phosphine oxides. Our experiments have shown that their analogues, secondary phosphine sulfides, under similar conditions provide exclusively the anti-Markovnikov adducts (Scheme 2). On the other hand, vinyl ethers and vinyl selenides (congeners of vinyl sulfides) were found to react with phosphine oxide **1a** at 80 °C for about 30 and 20 h, respectively, to deliver difficult-to-separate mixtures of organophosphorus compounds (^31^P NMR).

To gain a primary insight into the reaction mechanism, several experiments were carried out. On example of phosphine oxide **1a** and vinyl sulfide **2c**, we have shown that the reaction proceeds in the dark with the same efficiency as in the light. Therefore, the photochemical pathway of the reaction is hardly probable. Also, the reaction was established under an argon atmosphere. Under these conditions (argon, 80 °C for 18 h, exemplified by **1a**/**2c** pair) the formation of products **3a–h** does not take place and the starting phosphine oxide remained almost intact (^31^P NMR). This indicates that the reaction requires the presence of oxygen. In the other experiment, when TEMPO, a widely used radical scavenger, was added (10 mol %) into the reaction system **1a**/**2c**, the product **3d** was also formed, however, a longer reaction time was required for complete conversion of secondary phosphine oxide **1a** as compared to TEMPO-free conditions (15 vs 11 h). Meanwhile, this observation does not completely exclude a radical mechanism since the cross-coupling reactions between TEMPO and radical intermediates can be reversible [31]. In future, we intend to check various radical scavengers (other than TEMPO) in order to better understand the reaction mechanism.

**Scheme 3:**
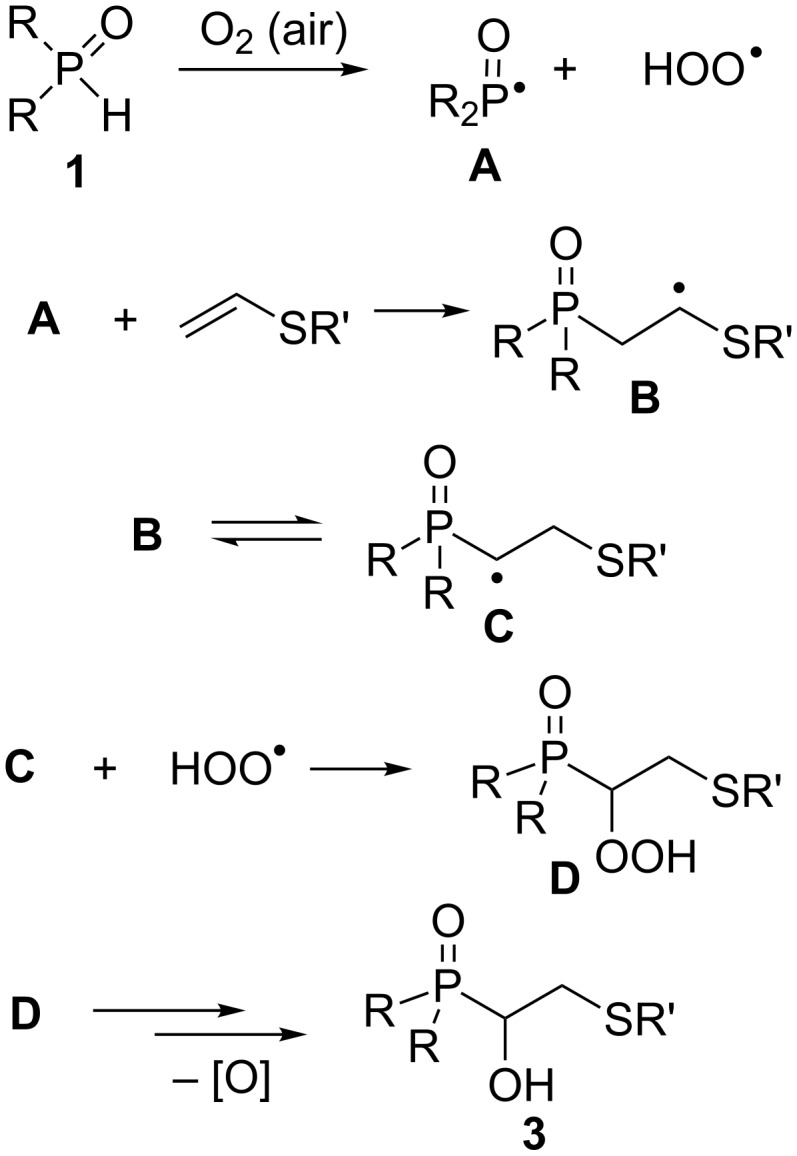
Putative mechanism.

Taking these data into account, the following mechanism is suggested (Scheme 3). The first step is assumed to be the generation of phosphinoyl (**A**) and hydroperoxyl (HOO^•^) radicals by the reaction of O_2_ with phosphine oxide **1**. Earlier, the transfer of a hydrogen atom from the P(O)H species to molecular oxygen has been reported for example for Ph_2_P(O)H [30]. Then, the radical addition of **A** to vinyl sulfide, proceeding in an anti-Markovnikov manner, takes place. Subsequently, a 1,2-intramolecular transfer of an H atom within the radical adduct **B** (from PCH_2_ group to radical center) leads to the formation of R_2_P(O)-stabilized radical **C**. The latter recombines with a hydroperoxide radical to afford the metastable hydroperoxide **D**, thermal decomposition of which give rise to the final product **3**.

Although quantum chemical computations [MP2/6-311++G(d,p)//B3LYP/6-311++G(d,p)] of the model radicals **B** and **C** (with R, R*'* = Me) reveals that the latter is energetically less preferred than the former, their energy difference is too small (4.38 kcal/mol) to completely prohibit the **B**→**C** transformation.

## Conclusion

In summary, we have disclosed an aerobic addition of secondary phosphine oxides to vinyl sulfides under solvent- and catalyst-free conditions, which provides an efficient approach to hitherto unknown 1-hydroxy-2-(organosulfanyl)ethyl(diorganyl)phosphine oxides in one step. The synthesized phosphine oxides, bearing hydroxy and sulfide functions, represent prospective building blocks for organic synthesis and interesting ligands for metal complexes. The results obtained contribute to the basic chemistry of both phosphine oxides and vinyl sulfides.

## Supporting Information

File 1 General remarks, experimental procedure and characterization data; crystallographic information for **3d**; ^1^H, ^13^C & ^31^P NMR spectra of synthesized compounds.

File 2 CIF file of compound **3d**.
