# Supplementary material for: Aerobic addition of secondary phosphine oxides to vinyl sulfides: a shortcut to 1-hydroxy-2-(organosulfanyl)ethyl(diorganyl)phosphine oxides
Source: Beilstein J Org Chem. 2015 Oct 23;11:1985–90. doi: 10.3762/bjoc.11.214 (PMC4660992; doi:10.3762/bjoc.11.214)

Supporting Information

for

Aerobic addition of secondary phosphine oxides to vinyl sulfides: a shortcut to 1-hydroxy-2-(organosulfanyl)ethyl(diorganyl)phosphine oxides

Svetlana F. Malysheva[1], Alexander V. Artem'ev[1], Nina K. Gusarova[1], Nataliya A. Belogorlova[1], Alexander I. Albanov[1], C. W. Liu[2] and Boris A. Trofimov*[1]

Address: [1]A. E. Favorsky Irkutsk Institute of Chemistry, Siberian Branch, Russian Academy of Sciences, 1 Favorsky Str., 664033 Irkutsk, Russian Federation and [2]Department of Chemistry, National Dong Hwa University, Hualien 97401, Taiwan

Email: Boris A. Trofimov - boris_trofimov@irioch.irk.ru

* Corresponding author

General remarks, experimental procedure and characterization data; crystallographic information for 3d; ^{1}H, ^{13}C & ^{31}P NMR spectra of synthesized compounds

Table of Contents

	Page
General information	S2
Crystallography	S2
Typical procedure	S3
Analytical data	S3
Computational details	S7
^{1}H, ^{13}C and ^{31}P NMR Spectra	S9

General information

The ^{1}H, ^{13}C and ^{31}P NMR spectra were recorded on a Bruker AV-400 spectrometer (400.13, 100.62 and 161.98 MHz, respectively) at ambient temperature (23–25 °C). Chemical shifts were reported in δ (ppm) relative to $CDCl_3$ residual peak (^{1}H, ^{13}C) as an internal standard or H_3PO_4 (^{31}P) as an external standard. FTIR spectra were obtained with a Bruker Vertex 70 spectrometer. The C, H microanalyses were performed on a Flash EA 1112 CHNS-O/MAS analyzer, while the P contents were determined by combustion method. Melting points (uncorrected) were established using a Kofler micro hot stage.

Vinyl sulfides **2a–c** were prepared by direct vinylation of corresponding thiols with acetylene in KOH/DMSO system.[1] Secondary phosphine oxides **1a–f** were synthesized by oxidation of the corresponding phosphines with H_2O_2 in acetone/water medium at ambient temperature. The initial phosphines were prepared from the corresponding styrenes or 2-vinylfurane and red phosphorus as described in the literature,[2] whereas diphenylphosphine was used as purchased (Alfa Aesar).

Crystallography

The single crystals of **3d** were obtained by slow evaporation of its solution in iPrOH. Crystals were formed within several days. Each single crystal was mounted on a glass fiber with epoxy resin. All reflection data were collected on a Bruker SMART APEX-II CCD instrument by using graphite monochromatic Mo Kα radiation (λ = 0.71073 Å). A semiempirical absorption correction by using SADABS[3] was applied, and the raw data frame integration was performed with SAINT.[4] Structures were solved by direct method and were refined against the least-squares methods on F^2 with the SHELXL-97 package,[5] incorporated in SHELXTL-PC V6.14.8.[6] All non-hydrogen atoms were refined anisotropically.

Crystal data for $C_{24}H_{27}O_2PS$ (M = 410.48 g/mol): monoclinic, space group $P2_1/c$ (no. 14), a = 14.4632(6) Å, b = 10.7632(5) Å, c = 14.5754(6)Å, β = 105.3574(13)°, V = 2187.94(16) Å^3, Z = 4, T = 296(2)K, μ(Mo Kα) = 0.238 mm^{-1}, D_{calc} = 1.246g/cm^3, 12846 reflections measured (4.766° ≤ 2Θ ≤ 50°), 3856 unique (R_{int} = 0.0546, R_{sigma}= 0.0579) which were used in all calculations. The final R_1 was 0.0448 (I >2σ(I)) and wR_2 was 0.1002 (all data).

---

[1]Gusarova, N. K.; Chernysheva, N. A.; Yas'ko, S. V.; Trofimov, B. A. *Russ. Chem. Bull.* **2013**, *62*, 438-440.

[2]Trofimov, B.A.; Brandsma, L.; Arbuzova, S. N.; Malysheva, S. F.; Gusarova, N. K. *Tetrahedron Lett.***1994**, *35*, 7647-7650.

[3]Sheldrick, G. M. SADABS, University of Gottingen, Gottingen, Germany, **1996**.

[4]SAINT V4.043: Software for the CCDC Detector System, Bruker Analystic X-ray System, Madison, WI, USA, **1995**.

[5]Sheldrick, G. M. *Acta Crystallogr., Sect. A.* **2008**, *64*, 112-122.

[6]*SHELXL 5.10 (PC version): Program Library for Structure Solution and molecular Graphics*, Bruker Analytical X-ray System, Madison, WI, USA, **1998**.

CCDC 1046604 contains the supplementary crystallographic data for this paper. These data can be obtained free of charge from The Cambridge Crystallographic Data Centre via www.ccdc.cam.ac.uk/data_request/cif.

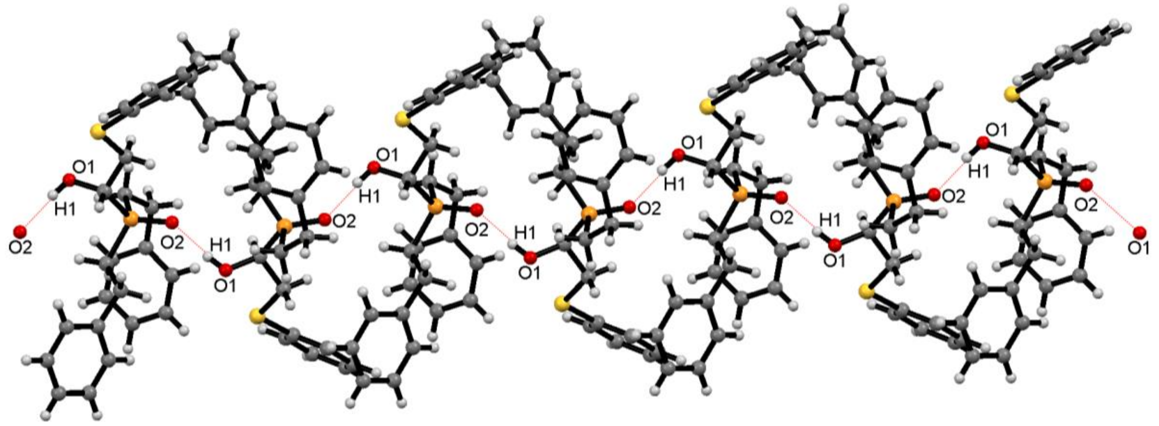

Figure S1. H-bonded 1D polymeric network of **3d** (along *b*-axis).

Typical procedure

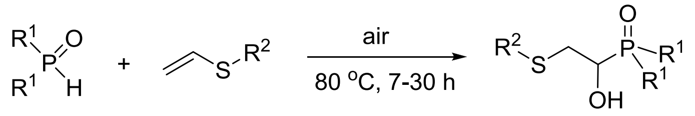

1a-f **2a-c** **3a-h** (70-93%)

A mixture of secondary phosphine oxide **1a–f** (1.0 mmol) and vinyl sulfide **2a–c** (1.1 mmol) was charged in 50 mL flask equipped with a reflux condenser and stirred at 80 °C for 7–30 h (see Table 1). After completion of the reaction (^{31}P NMR monitirong), the crude product was purified by washing with Et_2O (1 × 5 mL) followed by flash chromatography on basic Al_2O_3 (1 cm, $CHCl_3$ as eluent) to give phosphine oxides **3a-h**.

Analytical data

2-(*tert*-Butylthio)-1-(diphenethylphosphoryl)-1-ethanol (3a)

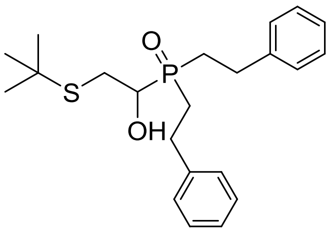

Yield: 312 mg (80%); light-beige powder, mp 110-115 °C (*i*-PrOH). IR (KBr, cm^{-1}): 3438, 3086, 3028, 2957, 2923, 2855, 1633, 1603, 1497, 1454, 1365, 1312, 1269, 1214, 1144, 1121, 1070, 1029, 1008, 937, 908, 834, 783, 750, 730, 697, 580, 508, 482, 430. ^{1}HNMR (400.13 MHz, $CDCl_3$): δ = 1.32 (s, 9 H, Me), 2.12-2.19 (m, 4 H, CH_2P), 2.78 (ddd, 1 H, $^2J_{HH}$ = 13.0, $^3J_{HH}$ = 11.1, $^3J_{PH}$ = 9.4 Hz, H_a in SC$\underline{H}_2$), 2.81-2.96 (m, 4H,

CH_2Ph), 3.19 (ddd, 1 H, $^2J_{HH}$ = 13.0, $^3J_{HH}$ = $^3J_{PH}$ = 3.8 Hz, H_b in $SC\underline{H}_2$), 3.94 (ddd, 1 H, $^3J_{HH}$= 11.1, $^3J_{HH}$ = 3.8, $^2J_{PH}$ = 3.3 Hz, $C\underline{H}P$), 4.52 (1 H, OH), 7.20-7.98 (m, 10H, Ph). ^{13}C NMR (100.62 MHz, $CDCl_3$): δ = 27.4 and 27.6 (d, $^2J_{PC}$ = 3.5, Hz, $\underline{C}H_2Ph$), 26.8 and 28.3 (d, $^1J_{PC}$ = 60.4 and $^1J_{PC}$ = 60.8 Hz, CH_2P), 30.7 (d, $^2J_{PC}$ = 1.7 Hz, CH_2S), 31.2 ($\underline{Me}_3C$), 43.4 ($\underline{C}Me_3$), 67.8 (d, $^1J_{PC}$ = 78.5 Hz, CHP), 126.6 (C_p, Ph), 128.2 and 128.2 (C_o, Ph), 128.8 and 128.8 (C_m, Ph), 134.0 (C_i, Ph), 141.2 and 141.1 (d, $^3J_{PC}$ = 13.8 Hz, C_i, Ph). ^{31}P NMR (161.98 MHz, $CDCl_3$): δ = 50.34. Anal. Calcd for $C_{22}H_{31}O_2PS$: C, 67.66; H, 8.00; P, 7.93. Found: C, 67.75; H, 8.08; P, 7.69.

1-(Diphenethylphosphoryl)-2-(heptylthio)-1-ethanol (3b)

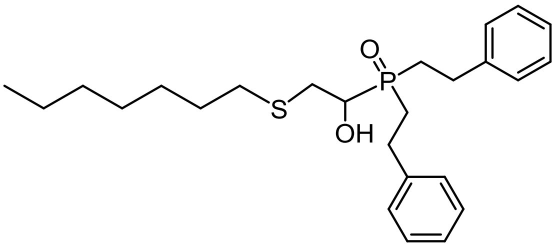

Yield: 337 mg (78%); light-yellow oil. IR (film, cm^{-1}): 3167, 3086, 3063, 3028, 2954, 2926, 2855, 1654, 1603, 1584, 1497, 1454, 1404, 1378, 1271, 1218, 1143, 1132, 1073, 1031, 1006, 972, 946, 910, 841, 783, 750, 699, 582. ^1H NMR (400.13 MHz, $CDCl_3$): δ = 0.86 (t, 3H, $^3J_{HH}$ = 7.1 Hz, Me), 1.24-1.34 [m, 8 H, $(C\underline{H}_2)_4Me$], 1.55-1.58 (m, 2 H, $C\underline{H}_2Am$), 2.51 (t, 2 H, $^3J_{HH}$ = 7.5 Hz, $CHCH_2SC\underline{H}_2$), 1.98-2.23 (m, 4 H, CH_2P), 2.89-2.98 (m, 4 H, $C\underline{H}_2Ph$), 2.76 (ddd, 1 H, $^2J_{HH}$ = 13.8, $^3J_{HH}$ = 11.2, $^3J_{PH}$ = 8.2 Hz, 1 H_a, $CHC\underline{H}_2SCH_2$), 3.11 (ddd, 1H, $^2J_{HH}$ = 13.8, $^3J_{HH}$ = $^3J_{PH}$ = 3.2 Hz, H_b, $CHCH_2SC\underline{H}_2$), 3.97 (ddd, 1 H, $^2J_{HH}$ = 11.2, $^3J_{HH}$ = 3.2, $^2J_{PH}$ = 3.5 Hz, CHP), 4.62 (1H, OH), 7.13-7.24 m (10 H, Ph). ^{13}C NMR (100.62 MHz, $CDCl_3$): δ = 13.8 (Me), 22.3 ($\underline{C}H_2Me$), 27.1 and 27.2 (d, $^2J_{PC}$= 3.4 Hz, $\underline{C}H_2Ph$), 26.4 and 27.8 (d, $^3J_{PC}$ = 58.6 Hz, CH_2P), 28.6 and 28.6 ($\underline{C}H_2Pr$ and $\underline{C}H_2Bu$), 29.4 ($\underline{C}H_2Am$), 31.5 ($\underline{C}H_2Et$), 31.9 ($CHCH_2S\underline{C}H_2$), 33.8 ($CH\underline{C}H_2SCH_2$), 66.8 (d, $^1J_{PC}$ = 73.3 Hz, CHP), 126.3 (C_p, Ph), 127.8 and 127.9 (C_o, Ph), 128.4 (C_m, Ph), 140.7 and 140.8 (d, $^3J_{PC}$ = 12.9 Hz, C_i, Ph). ^{31}P NMR (161.98 MHz, $CDCl_3$): δ = 50.78. Anal. Calcd for $C_{25}H_{37}O_2PS$: C, 69.41; H, 8.62; P, 7.16. Found: C, 69.32; H, 8.50; P, 7.01.

1-(Diphenylphosphoryl)-2-(phenylthio)-1-ethanol (3c)

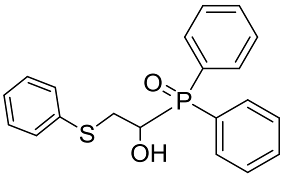

Yield: 248 mg (70%); light-beige powder, mp 168-170 ^oC (*i*-PrOH). IR (KBr, cm^{-1}): 3437, 3076, 3054, 2956, 2920, 2853, 1589, 1580, 1478, 1438, 1185, 1119, 1088, 1070, 1024, 998, 983, 747, 729, 719, 693, 552, 530, 512. ^1H NMR (400.13 MHz, $CDCl_3$): δ = 2.95 (ddd, 1 H, $^2J_{HH}$ = 14.3, $^3J_{HH}$ = 11.5, $^3J_{PH}$ = 7.5 Hz, CH_a in CH_2S), 3.56 (ddd, 1 H, $^2J_{HH}$ = 14.3, $^3J_{HH}$ = $^3J_{PH}$ = 2.7 Hz, H_b in SCH_2), 4.37 (ddd, 1 H, $^3J_{HH}$ = 11.5, $^3J_{HH}$ = $^2J_{PH}$ = 2.7 Hz, CHP), 7.21 (m, 5 H, SPh), 7.45 (m, 4 H, H_m, PPh), 7.53 (m, 2 H, H_p, PPh), 7.79-7.80 and 7.82-7.86 (m, 4H_o, PPh). ^{13}C

NMR (100.62 MHz, $CDCl_3$): δ = 36.6 (d, $^4J_{PC}$ = 4.7 Hz, CH_2S), 68.2 (d, $^1J_{PC}$ = 83.2 Hz, PCH), 127.06 (C_p, PhS), 128.5 (d, $^3J_{PC}$ = 12.1 Hz, C_m, PhP), 129.2 (C_p, PhS), 130.6 (C_o, PhS), 131.4 (d, $^3J_{PC}$ = 9.1 Hz, C_o, PhP), 132.2 (C_m, PhP), 133.7 (d, C_i, PhS), 129.1 (d, $^3J_{PC}$ = 80.0 Hz, C_i, PhP). ^{31}P NMR (161.98 MHz, $CDCl_3$): δ = 31.39. Anal. Calcd for $C_{20}H_{19}O_2PS$: C, 67.78; H, 5.40; P, 8.74. Found: C, 67.65; H, 5.56; P, 8.58.

1-(Diphenethylphosphoryl)-2-(phenylthio)ethanol (3d)

Yield: 374 mg (91%); Colorless crystals, mp 109-111 ^oC (*i*-PrOH). IR (KBr, cm^{-1}): 3429, 3084, 3024, 2960, 2923, 2867, 2851, 1602, 1582, 1496, 1473, 1455, 1438, 1417, 1391, 1311, 1269, 1212, 1142, 1122, 1069, 1026, 1009, 939, 912, 866, 808, 745, 697, 577, 473, 460. ^1H NMR (400.13 MHz, $CDCl_3$): δ = 2.03-2.21 (m, 4 H, CH_2P), 2.86-2.94 (m, 4H, $C\underline{H}_2Ph$), 3.08 (ddd, 1 H, $^2J_{HH}$ = 13.8, $^3J_{HH}$ = 11.1, $^3J_{PH}$ = 7.3 Hz, H_a in SCH_2), 3.56 (ddd, 1 H, $^2J_{HH}$ = 13.8, $^3J_{HH}$ = $^3J_{PH}$ = 3.2 Hz, H_b in SCH_2), 3.95 (ddd, 1 H, $^3J_{HH}$ = 11.1, $^3J_{HH}$ = 3.2, $^2J_{PH}$ = 3.5 Hz, CHP), 4.07 (1 H, OH), 7.13-7.36 (m, 15 H, Ph). ^{13}C NMR (100.62 MHz, $CDCl_3$): δ = 27.3 and 27.5 (d, $^2J_{PC}$ = 3.4 Hz, $\underline{C}H_2Ph$), 26.6 and 28.1 (d, $^1J_{PC}$ = 60.5 Hz, CH_2P), 36.4 (d, $^2J_{PC}$ = 4.2 Hz, $\underline{C}H_2SPh$), 66.8 (d, $^1J_{PC}$ = 77.7 Hz, CHP), 126.5 and 126.5 (C_p, $PhCH_2$), 128.1 and 128.1 (C_o, $PhCH_2$), 128.68 and 128.71 (C_m, $PhCH_2$), 127.0 (C_p, PhS), 129.3 (C_o, PhS), 130.4 (C_m, PhS), 134.2 (C_i, PhS), 140.9 and 141.0 (d, $^3J_{PC}$ = 13.4 Hz, C_i, $PhCH_2$). ^{31}P NMR (161.98 MHz, $CDCl_3$): δ = 50.24. Anal. Calcd for $C_{24}H_{27}O_2PS$: C, 70.22; H, 6.63; P, 7.55. Found: C, 70.14; H, 6.78; P, 7.36.

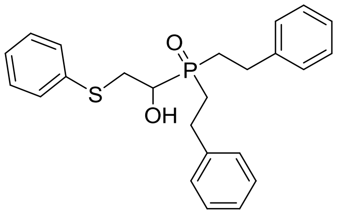

1-[Bis(4-methylphenethyl)phosphoryl]-2-(phenylthio)ethanol (3e)

Yield: 408 mg (93%); White powder, mp 107-109 ^oC (hexane). IR (KBr, cm^{-1}): 3165, 3051, 3020, 2921, 2864, 1583, 1515, 1481, 1439, 1414, 1379, 1312, 1270, 1219, 1145, 1129, 1070, 1024, 1001, 938, 872, 848, 808, 742, 691, 539, 528, 484, 473. ^1H NMR (400.13 MHz, $CDCl_3$): δ = 1.99-2.15 (m, 4 H, $C\underline{H}_2P$), 2.27 and 2.78 (s, 6H, Me), 2.82-2.85 (m, 4H, $C\underline{H}_2C_6H_4$), 3.09 (ddd, 1 H, $^2J_{HH}$ = 13.9, $^3J_{HH}$ = 11.1, $^3J_{PH}$ = 7.7 Hz, H_a in $SC\underline{H}_2$), 3.54 (ddd, 1 H, $^2J_{HH}$ = 13.9, $^3J_{HH}$ = $^3J_{PH}$ = 3.2 Hz, H_b in $SC\underline{H}_2$), 3.96 (ddd, 1 H, $^3J_{HH}$ = 11.1, $^3J_{HH}$ = $^2J_{PH}$ = 3.2 Hz, CHP), 7.02 -7.37 (m, 13 H, Ph). ^{13}C NMR (100.62 MHz, $CDCl_3$): δ = 21.0 (Me), 26.9 and 27.1 (d, $^2J_{PC}$ = 3.5 Hz, $\underline{C}H_2C_6H_4$), 26.8 and 28.3 (d,$^1J_{PC}$ = 59.5 and $^1J_{PC}$ = 59.9 Hz, CH_2P), 36.5 (d, $^2J_{PC}$ = 3.9 Hz, $\underline{C}H_2S$), 67.0 (d, $^1J_{PC}$ = 76.3 Hz, PCH), 127.1 (C_p, Ph), 128.0 ($C_{2,6}$, C_6H_4), 129.3 (C_o, Ph), 129.4 ($C_{3,5}$, C_6H_4), 130.4 (C_m, Ph), 134.2 (C_i, SPh),

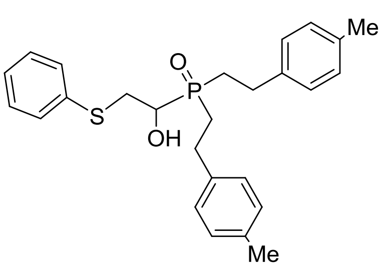

136.0 (C_4, C_6H_4), 137.9 and 138.0 (d, $^3J_{PC}$ = 12.5 and $^3J_{PC}$ = 12.9 Hz, C_1, C_6H_4). ^{31}P NMR (161.98 MHz, $CDCl_3$): δ = 51.01. Anal. Calcd for $C_{26}H_{31}O_2PS$: C, 71.20; H, 7.12; P, 7.06. Found: C, 71.19; H, 7.23; P, 6.89.

1-[Bis(4-*tert*-butylphenethyl)phosphoryl]-2-(phenylthio)ethanol (3f)

Yield: 470 mg (90%); Colorless crystals, mp 138-140 ^{o}C (*i*-PrOH). IR (KBr, cm^{-1}): 3396, 3078,

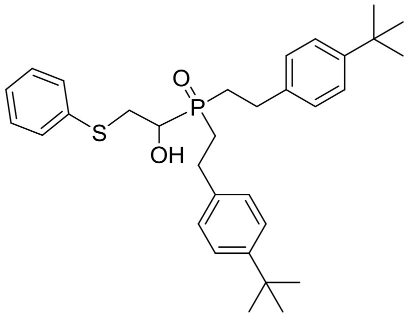

3058, 2959, 2903, 2865, 1584, 1517, 1509, 1481, 1439, 1414, 1398, 1362, 1314, 1270, 1216, 1188, 1147, 1125, 1010, 1063, 1025, 997, 876, 819, 779, 737, 688, 560, 517, 491, 473. ^{1}H NMR (400.13 MHz, $CDCl_3$): δ = 1.33, 1.34 (s, 18 H, Me), 2.08-2.25 (m, 4 H, CH_2P), 2.89-2.95 (m, 4H, $C\underline{H}_2C_6H_4$), 3.16 (ddd, 1 H, $^2J_{HH}$ = 13.9, $^3J_{HH}$ = 11.1, $^3J_{PH}$ = 8.1 Hz, H_a in $SC\underline{H}_2$), 3.62 (ddd, 1 H, $^2J_{HH}$ = 13.9, $^3J_{HH}$ = $^3J_{PH}$ = 3.2 Hz, H_b in $SC\underline{H}_2$), 4.04 (ddd, 1 H, $^3J_{HH}$ = 11.1, $^3J_{HH}$ = $^2J_{PH}$ = 3.1 Hz, CHP), 4.10 (1 H, OH), 7.13-7.46 (m, 13H, Ph, C_6H_4). ^{13}C NMR (100.62 MHz, $CDCl_3$): δ = 26.8, 27.0 (d, $^2J_{PC}$ = 3.2, $^2J_{PC}$ = 3.1 Hz, $\underline{C}H_2C_6H_4$), 26.8 and 28.2 (d, $^1J_{PC}$ = 59.7 Hz, $P\underline{C}H_2$), 31.4 ($\underline{Me_3}C$), 34.4 ($Me_3\underline{C}$), 36.6 (d, $^2J_{PC}$ = 4.2 Hz, CH_2S), 66.8 (d, $^1J_{PC}$ = 76.7 Hz, PCH), 125.6 ($C_{2,6}$, C_6H_4), 127.2 (C_p, Ph), 127.8 ($C_{3,5}$, C_6H_4), 129.4 (C_o, Ph), 130.6 (C_m, Ph), 134.0 (C_i, Ph), 137.8 and 137.9 (d, $^3J_{PC}$ = 13.3 Hz, C_1, C_6H_4), 149.4 (C_4, C_6H_4). ^{31}P NMR (161.98 MHz, $CDCl_3$): δ = 50.34. Anal. Calcd for $C_{32}H_{43}O_2PS$: C, 73.53; H, 8.29; P, 5.93. Found: C, 73.39; H, 8.27; P, 5.78.

1-[Bis(4-chlorophenethyl)phosphoryl]-2-(phenylthio)ethanol (3g)

Yield: 393 mg (82%); light-yellow oil. IR (film, cm^{-1}): 3159, 3062, 2921, 2851, 1597, 1583,

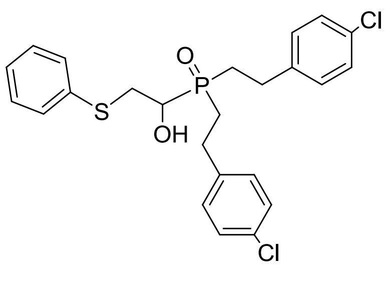

1492, 1439, 1408, 1220, 1144, 1133, 1093, 1073, 1015, 973, 847, 808, 740, 692, 657, 517. ^{1}H NMR (400.13 MHz, $CDCl_3$): δ = 1.90-2.16 (m, 4 H, $C\underline{H}_2P$), 2.85-2.89 (m, 4H, $C\underline{H}_2C_6H_4$), 3.06 and 3.55 (ddd, 2 H, $^2J_{HH}$ = 14.0, $^3J_{HH}$ = 10.9, $^3J_{PH}$ = 8.6, $^3J_{HH}$ = 3.2 Hz, $H_{a,b}$ in $SC\underline{H}_2$), 3.90 (ddd, 1 H, $^3J_{HH}$ = 10.9, $^3J_{HH}$ = $^2J_{PH}$ = 3.2 Hz, CHP), 4.00 (s, 1 H, OH), 7.07-7.39 (m, 13 H, Ph). ^{13}C NMR (100.62 MHz, $CDCl_3$): δ = 26.7 and 26.8 (d, $^2J_{PC}$ = 3.9 and $^2J_{PC}$ = 3.5 Hz, CH_2Ph), 26.7 and 28.2 (d, $^1J_{PC}$ = 60.5 and $^1J_{PC}$ = 60.4 Hz, CH_2P), 36.7 (d, $^2J_{PC}$ = 2.2 Hz, $\underline{C}H_2S$), 66.6 (d, $^1J_{PC}$ = 78.5 Hz, PCH), 127.5 (C_p, Ph), 128.9 ($C_{2,6}$, C_6H_4), 129.42 (C_o, Ph), 129.4 ($C_{3,5}$, C_6H_4), 130.8 (C_m, Ph), 132.4 (C_4, C_6H_4), 133.2 (C_i, SPh), 139.1 (d, $^3J_{PC}$ = 12.5 Hz, C_1, C_6H_4). ^{31}P NMR (161.98 MHz, $CDCl_3$): δ = 50.52. Anal. Calcd for $C_{24}H_{25}Cl_2O_2PS$: C, 60.13; H, 5.26; P, 6.46. Found: C, 60.02; H, 5.10; P, 6.33.

1-[Bis(2-(furan-2-yl)ethyl)phosphoryl]-2-(phenylthio)ethanol (3h)

Yield: 347 mg (89%); light-yellow oil. IR (film, cm^{-1}): 3188, 3149, 3119, 2954, 2922, 2853, 1716, 1596, 1584, 1507, 1481, 1439, 1407, 1383, 1339, 1236, 1215, 1148, 1072, 1025, 1008, 937, 915, 884, 805, 738, 693, 599, 474. ^{1}H NMR (400.13 MHz, $CDCl_3$): δ = 2.00-2.25 (m, 4 H, PCH_2), 2.86-2.95 (m, 4 H, CH_2Fur), 3.06 (ddd, 1 H, $^2J_{HH}$ = 13.6, $^3J_{HH}$ = 11.2, $^3J_{PH}$ = 7.6 Hz, H_a in CH_2S), 3.49 (ddd, 1 H, $^2J_{HH}$ = 13.6, $^3J_{HH}$ = $^3J_{PH}$ = 3.2 Hz, H_b in CH_2S), 3.99 (ddd, 1 H, $^2J_{HH}$ = 11.2, $^3J_{HH}$ = 3.2, $^2J_{PH}$ = 3.6 Hz, CHP), 5.99 (dd, 2 H, $^3J_{3\text{-}4}$ = 3.1 Hz, H_3 in Fur), 6.22 (dd, 2 H, $^3J_{4\text{-}3}$ = 3.2, $^3J_{4\text{-}5}$ = 2.0 Hz, H_4 in Fur), 7.30 (d, 2 H, $^3J_{5\text{-}4}$ = 1.9, $^3J_{5\text{-}3}$ = 0.8 Hz, H_5 in Fur), 7.18-7.27 (m, 5 H, Ph). ^{13}C NMR (100.62 MHz, $CDCl_3$): δ = 20.1 and 20.3 (d, $^2J_{PC}$ = 2.3 Hz, CH_2Fur), 23.3 and 24.7 (d, $^1J_{PC}$ = 60.4 and $^1J_{PC}$ = 60.8 Hz, PCH_2), 36.3 (d, $^2J_{PC}$ = 3.8 Hz, CH_2S), 67.0 (d, $^1J_{PC}$ = 78.8 Hz, PCH), 105.7 (C_3, Fur), 110.4 (C_4, Fur), 127.1 (C_p, Ph), 129.3 (C_o, Ph), 130.5 (C_m, Ph), 134.2 (C_i, Ph), 141.4 and 141.5 (C_5, Fur), 154.0 and 154.1 (d, $^3J_{PC}$ = 13.7 Hz, C_2, Fur). ^{31}P NMR (161.98 MHz, $CDCl_3$): δ = 50.53. Anal. Calcd for $C_{20}H_{23}O_4PS$: C, 61.53; H, 5.94; P, 7.93. Found: C, 61.39; H, 6.04; P, 7.78.

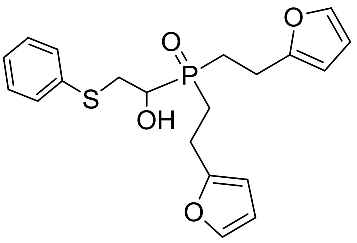

Computational details

All the computations were performed with the Gaussian 09 package.[7] The equilibrium geometries of radical **B** and **C** (where R = R' = Me; see Scheme 1) were calculated using B3LYP functional[8] and 6-311+G(d,p) basis set. The energies of the obtained structures were refined using the same basis set with the correlation effects included in the Møller–Plesset second order perturbation theory.

[7]Frisch M. J. et al., Gaussian 09, Revision A01, Gaussian, Inc., Wallingford, 2009.
[8]Becke A. D. *J. Chem. Phys.* **1993**, *98*, 5648-5652.

Structural data for radical ***B*** *from UMP2/6-311++G(d,p)//UB3LYP/6-311++G(d,p) calculations*

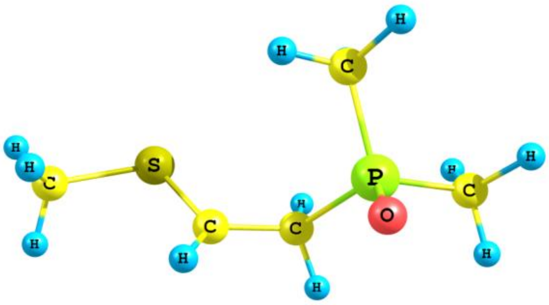

6	0.210778000	-0.731283000	-0.864370000
1	0.541092000	-1.740933000	-1.141113000
1	0.155640000	-0.142173000	-1.786273000
6	-1.100045000	-0.789695000	-0.163996000
1	-1.209829000	-1.392425000	0.728885000
15	1.584433000	-0.067183000	0.204359000
16	-2.325125000	0.389982000	-0.515706000
6	-3.669352000	-0.154669000	0.590842000
1	-4.495857000	0.541263000	0.448945000
1	-3.992124000	-1.161658000	0.326085000
1	-3.343795000	-0.121842000	1.630873000
6	3.056761000	-0.081248000	-0.885467000
1	3.900517000	0.350451000	-0.343057000
1	3.302882000	-1.115103000	-1.135714000
1	2.891355000	0.485475000	-1.805075000
6	1.197668000	1.700032000	0.466467000
1	2.002636000	2.148154000	1.052734000
1	1.093651000	2.238349000	-0.478563000
1	0.266979000	1.782289000	1.029382000
8	1.768438000	-0.845330000	1.474744000

E(MP2)= -1010.73428795a.u.

Structural data for radical ***C*** *from UMP2/6-311++G(d,p)//UB3LYP/6-311++G(d,p) calculations*

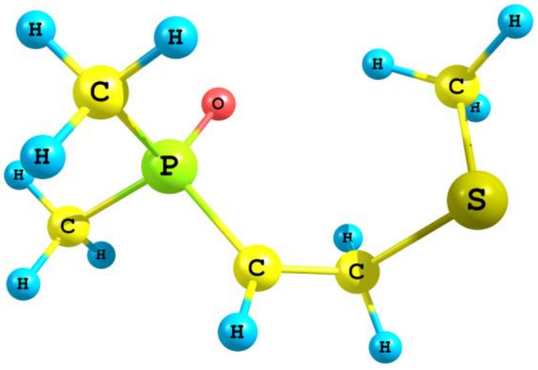

6	0.162946000	1.163341000	0.329344000
1	0.332311000	1.757912000	1.223388000
6	-1.121680000	1.317416000	-0.381658000
1	-1.486263000	2.344675000	-0.363799000
1	-1.083385000	0.961151000	-1.411001000
15	1.332233000	-0.140103000	-0.084409000
8	0.803030000	-1.084394000	-1.130207000
6	2.873225000	0.712838000	-0.587050000
1	3.210792000	1.418287000	0.175869000
1	3.650970000	-0.034900000	-0.760385000
1	2.688995000	1.249997000	-1.518773000
6	1.759348000	-0.947838000	1.502495000
1	2.537250000	-1.693371000	1.323005000
1	2.115792000	-0.222384000	2.237778000
1	0.871309000	-1.447498000	1.892330000
16	-2.443975000	0.298730000	0.489042000
6	-2.459543000	-1.211847000	-0.547171000
1	-3.029763000	-1.958268000	0.007370000
1	-2.954481000	-1.013878000	-1.498860000
1	-1.443437000	-1.568155000	-0.719566000

E(MP2)= -1010.72729933 a.u.

^{1}H, ^{13}C and ^{31}P NMR spectra

^{1}H NMR spectrum of phosphine oxide **3a** ($CDCl_3$)

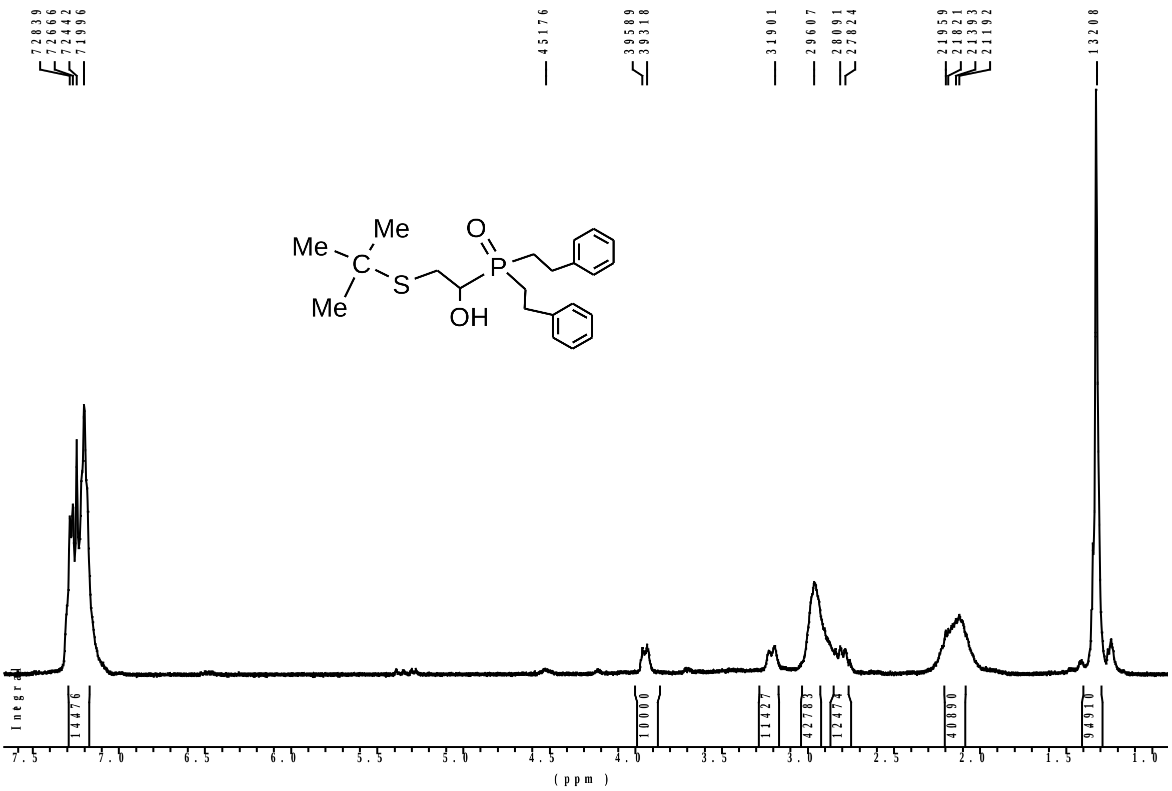

^{13}C NMR spectrum of phosphine oxide **3a** ($CDCl_3$)

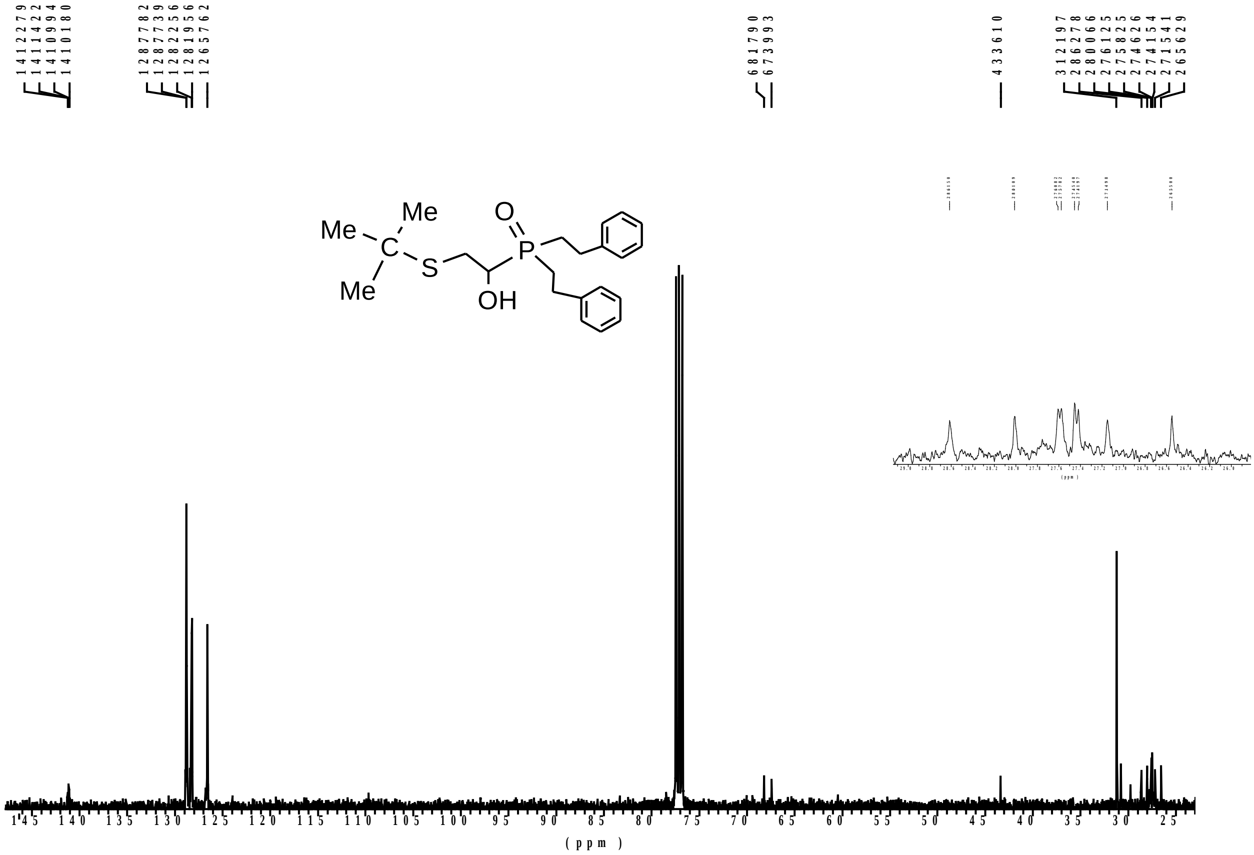

^{31}P NMR spectrum of phosphine oxide **3a** ($CDCl_3$)

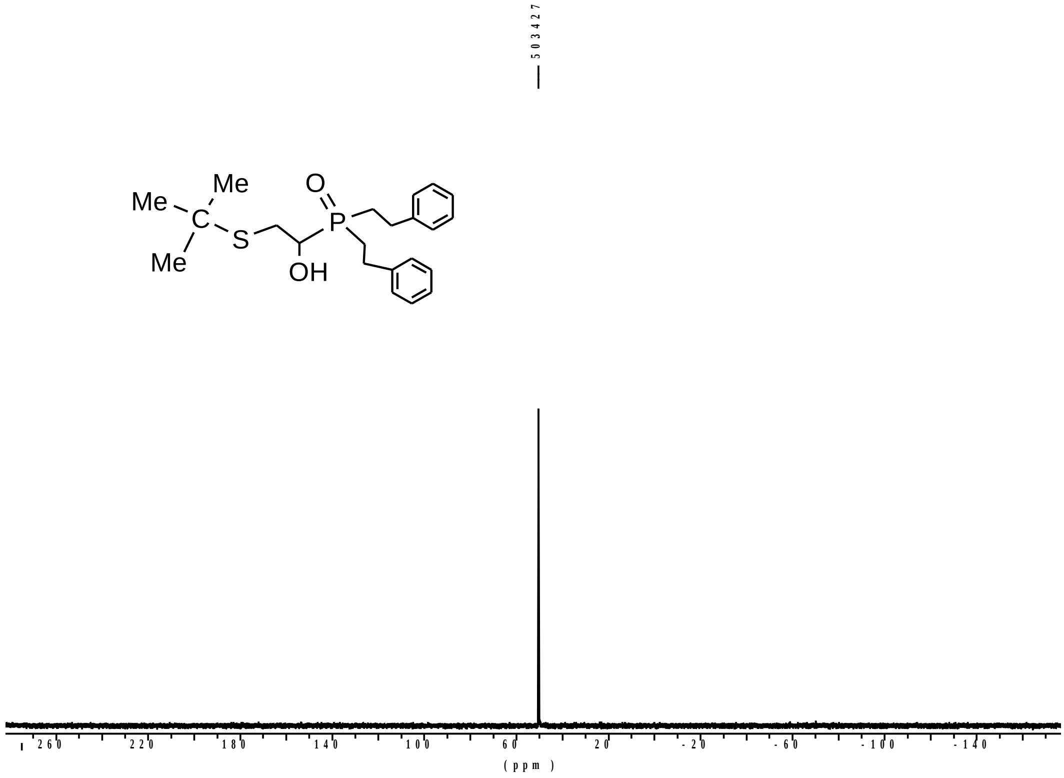

^{1}H NMR spectrum of phosphine oxide **3b** ($CDCl_3$)

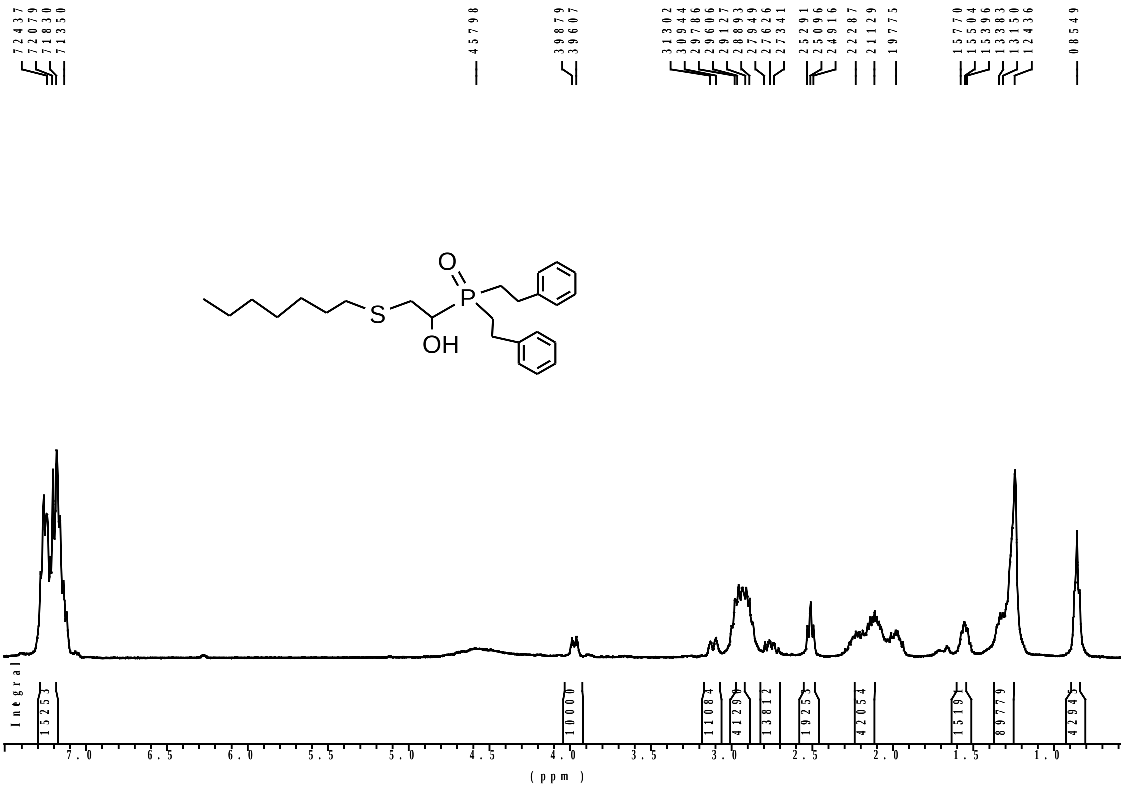

^{13}C NMR spectrum of phosphine oxide **3b** ($CDCl_3$)

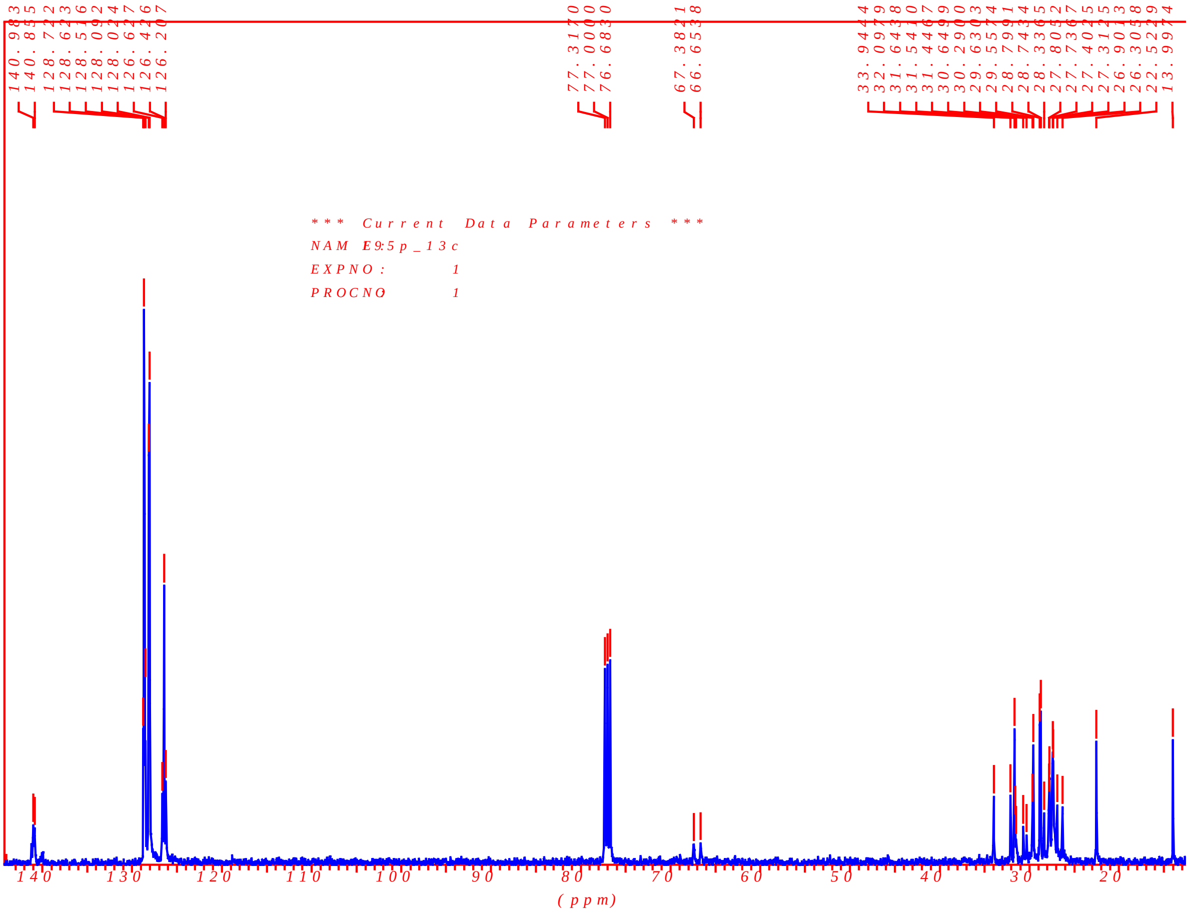

^{31}P NMR spectrum of phosphine oxide **3b** ($CDCl_3$)

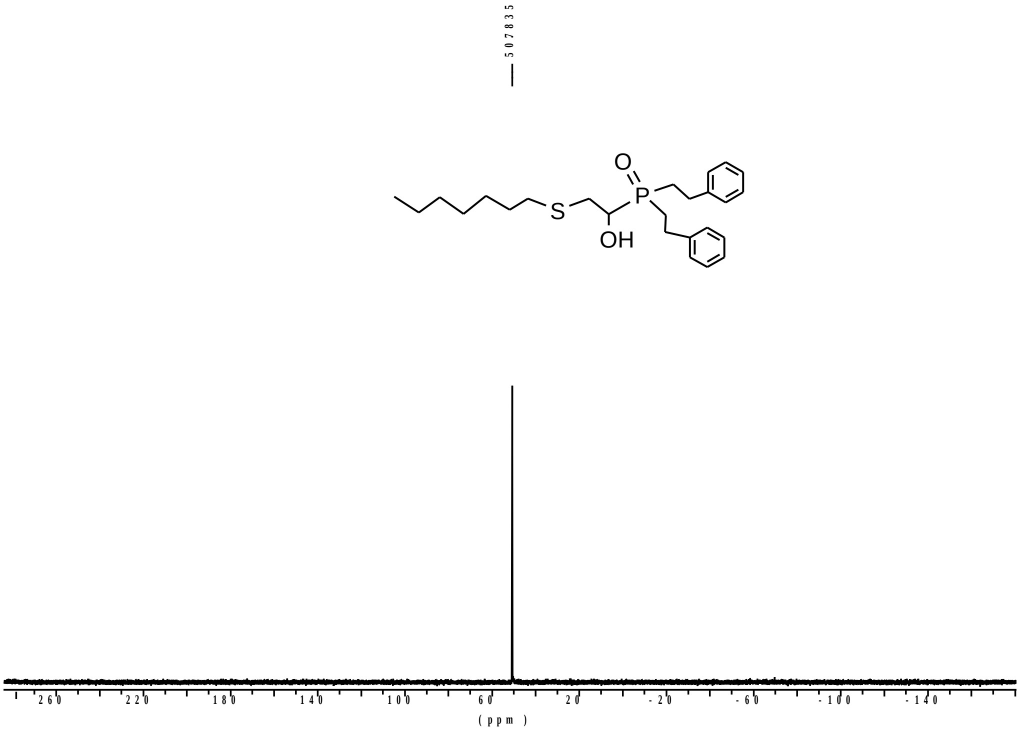

^{1}H NMR spectrum of phosphine oxide **3c** ($CDCl_3$)

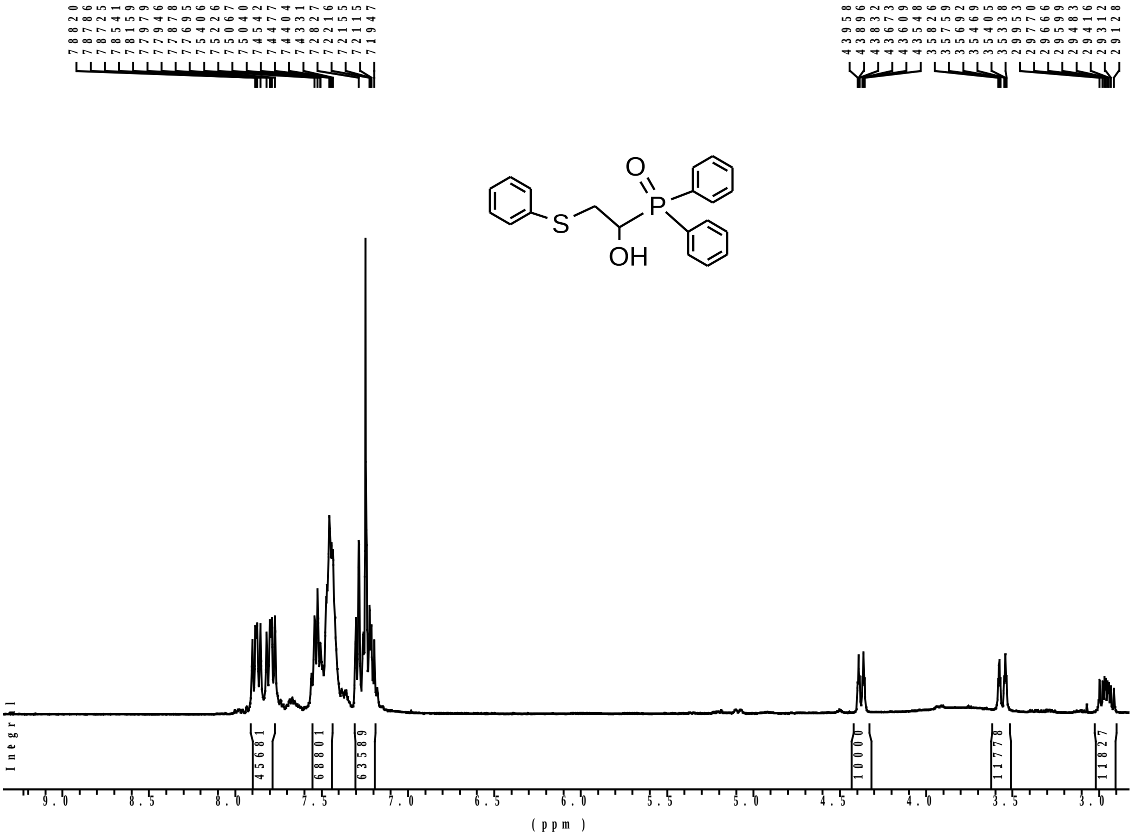

^{13}C NMR spectrum of phosphine oxide **3c** ($CDCl_3$)

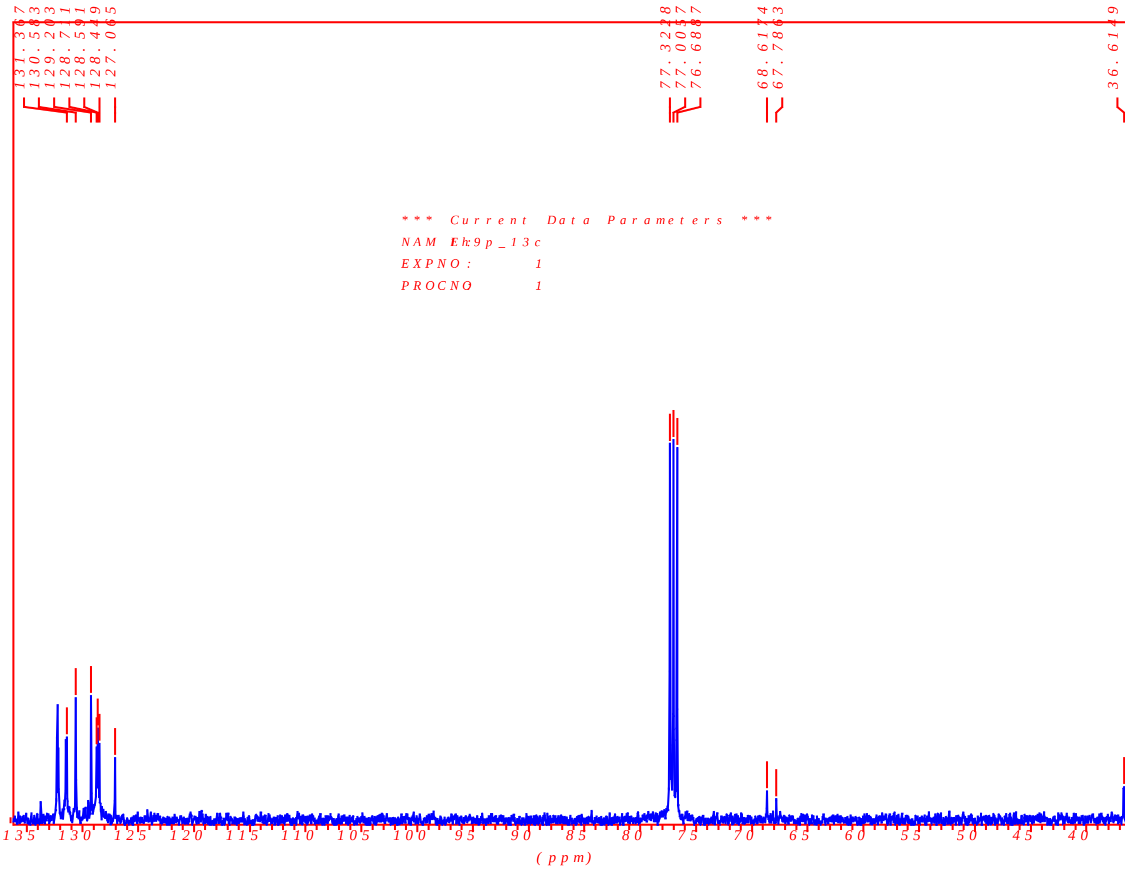

^{31}P NMR spectrum of phosphine oxide **3c** ($CDCl_3$)

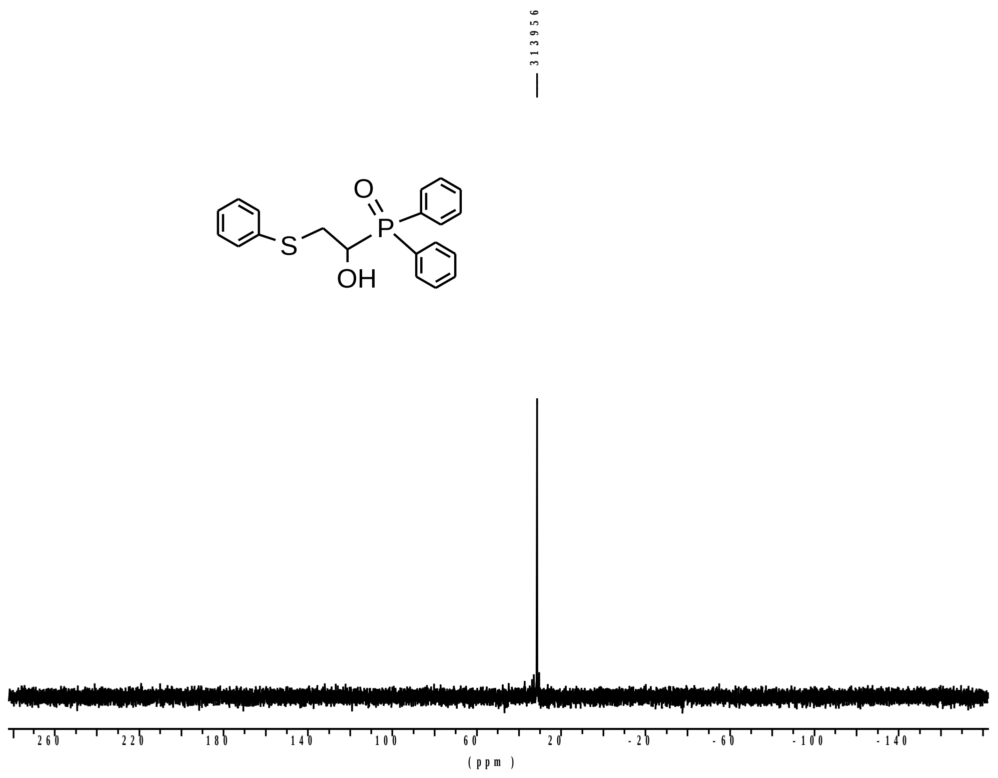

^{1}H NMR spectrum of phosphine oxide **3d** ($CDCl_3$)

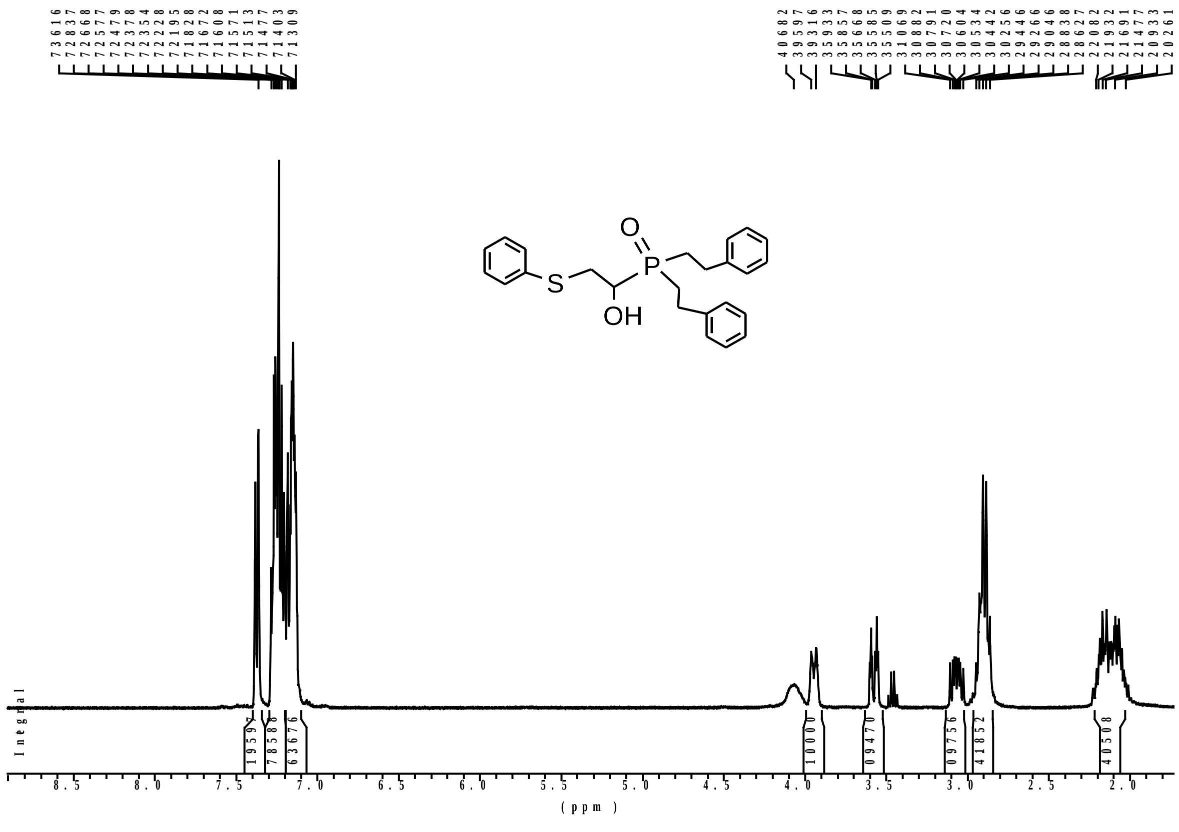

^{13}CjmodNMR spectrum of phosphine oxide **3d** ($CDCl_3$)

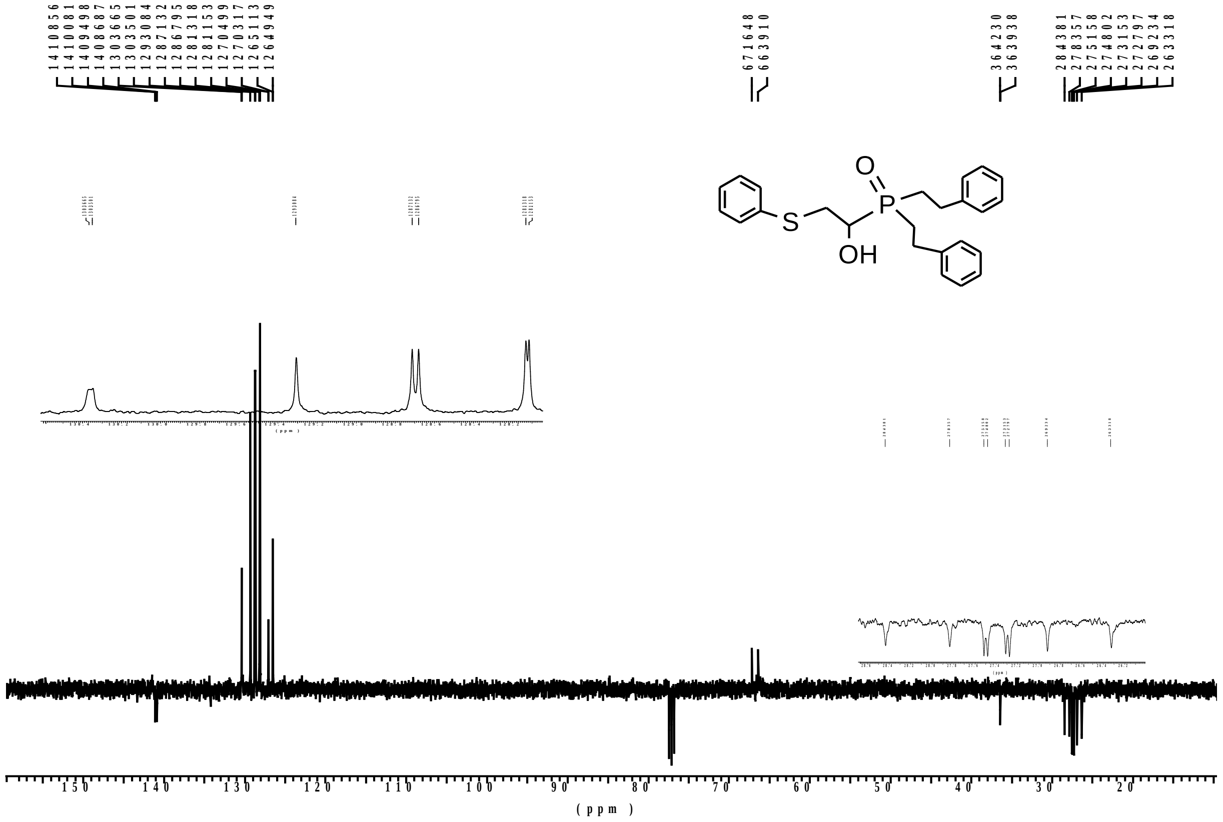

^{31}P NMR spectrum of phosphine oxide **3d** ($CDCl_3$)

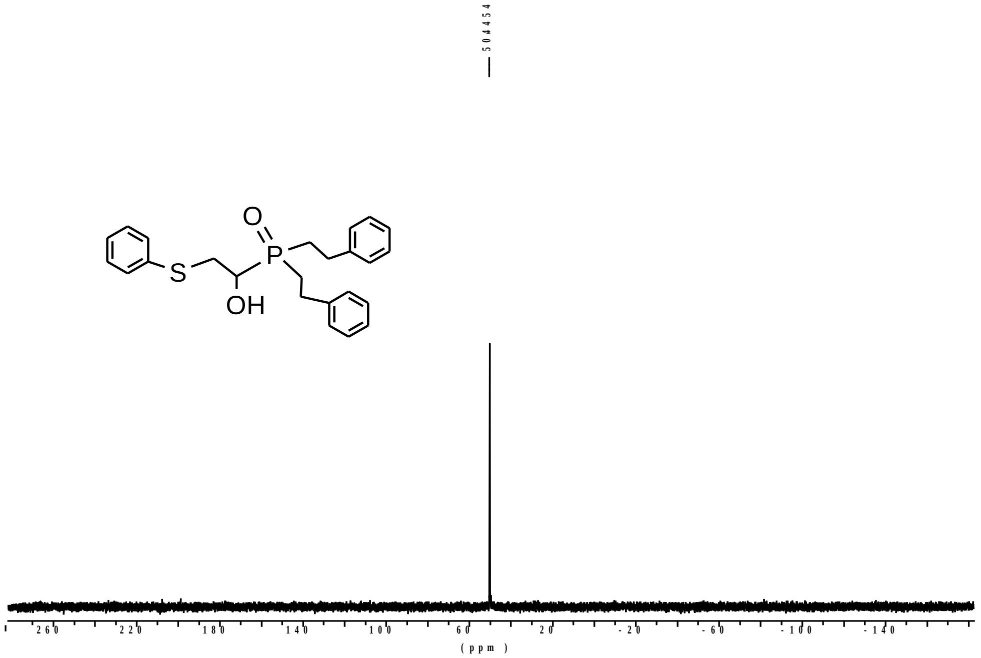

^{1}H NMR spectrum of phosphine oxide **3e** ($CDCl_3$)

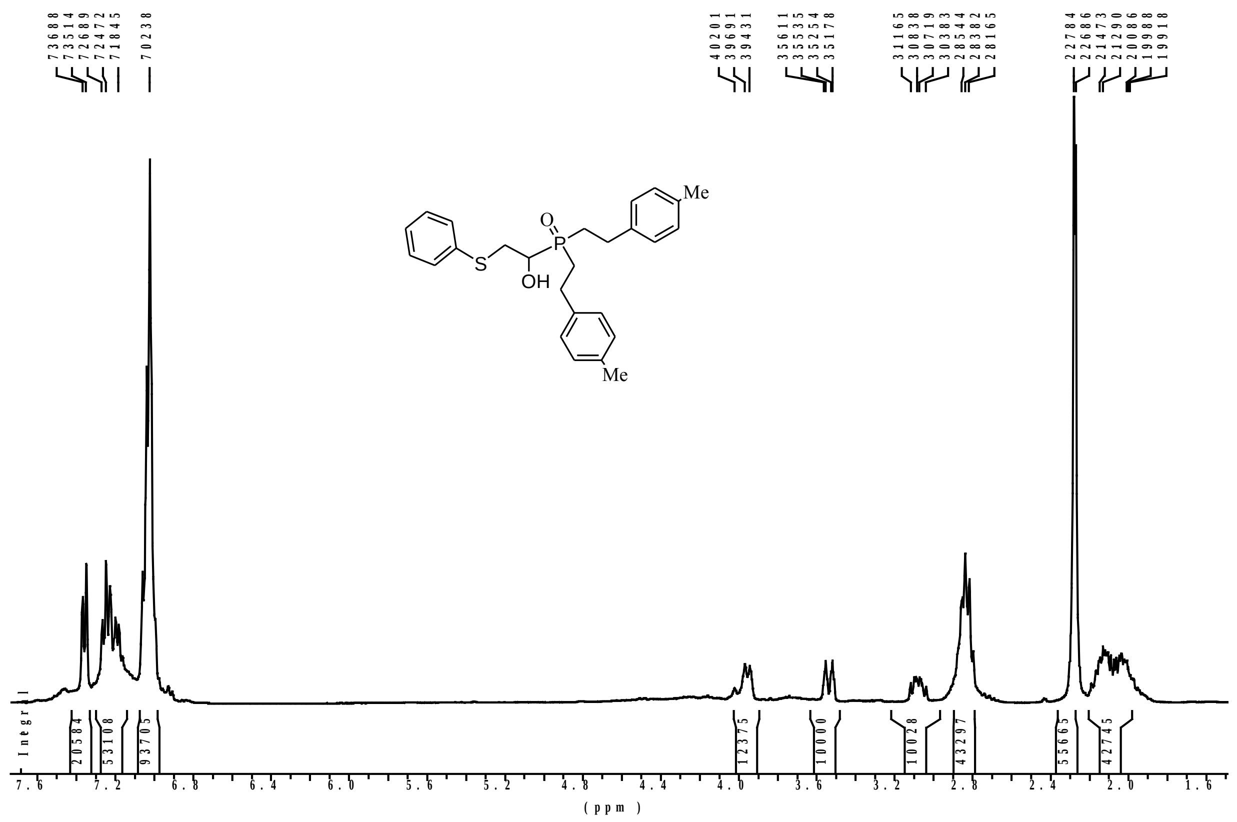

^{13}C NMR spectrum of phosphine oxide **3e** ($CDCl_3$)

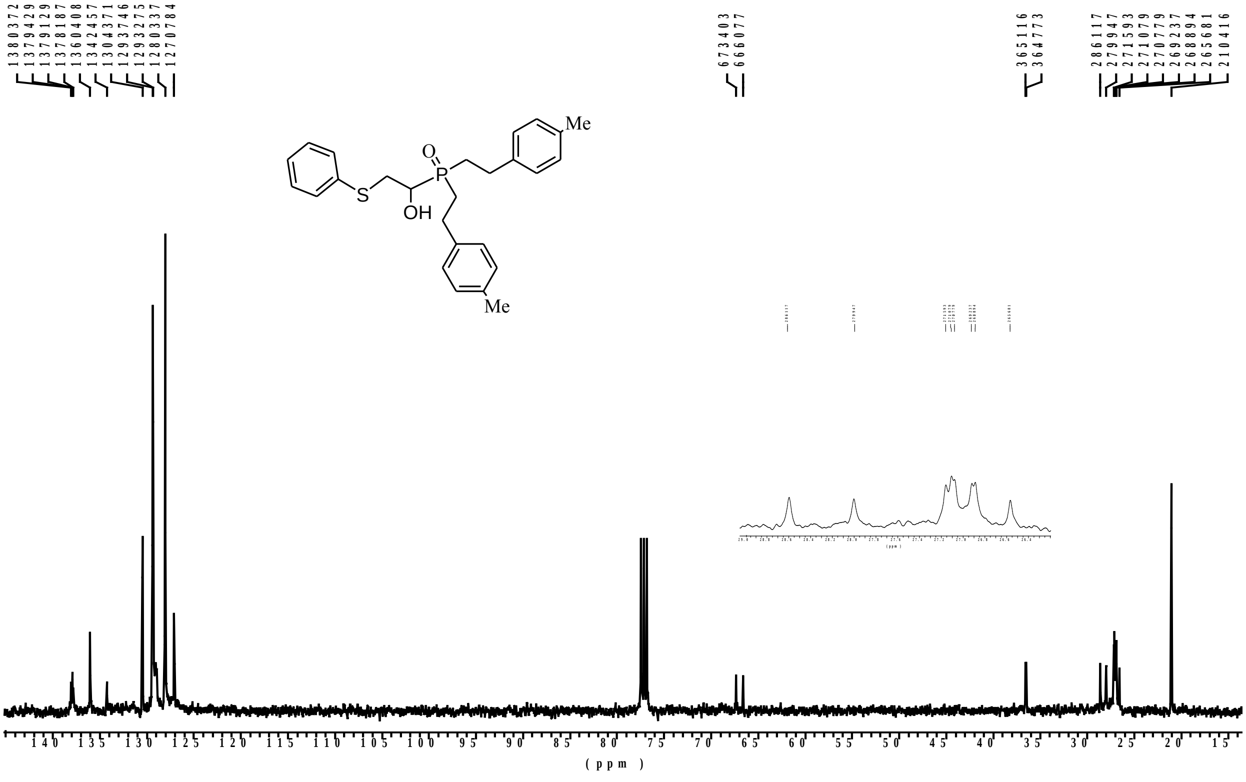

^{31}P NMR spectrum of phosphine oxide **3e** ($CDCl_3$)

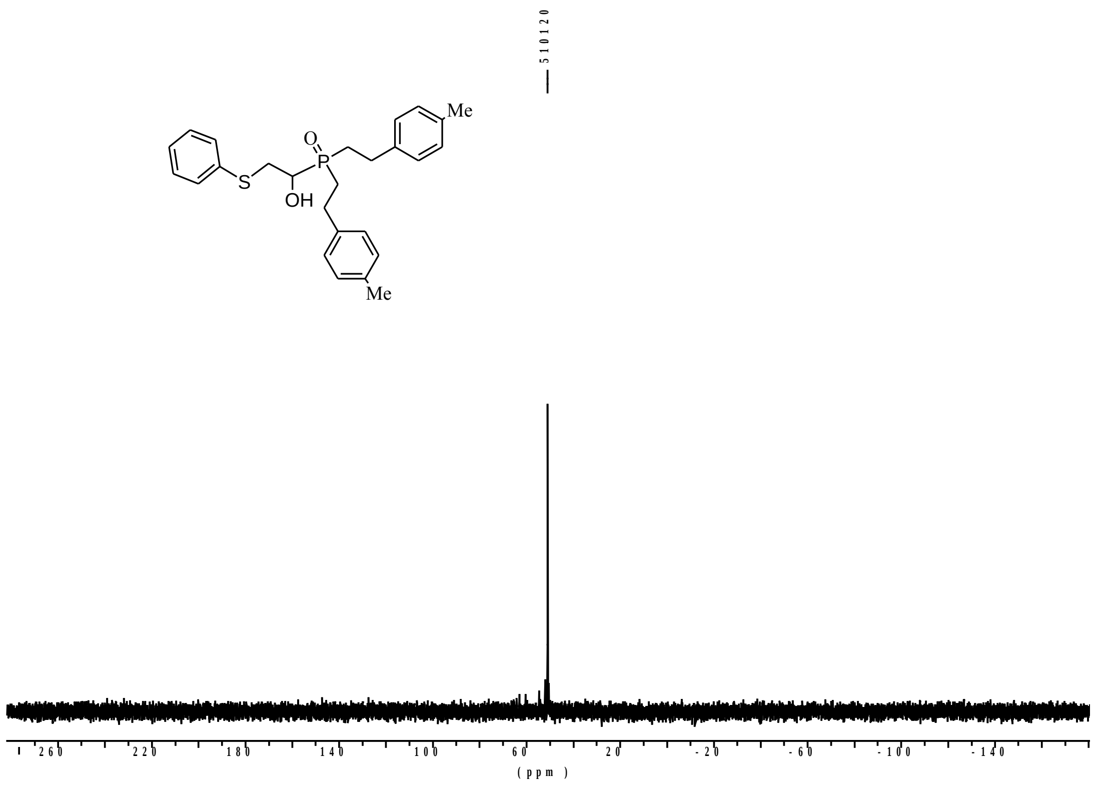

^{1}H NMR spectrum of phosphine oxide **3f** ($CDCl_3$)

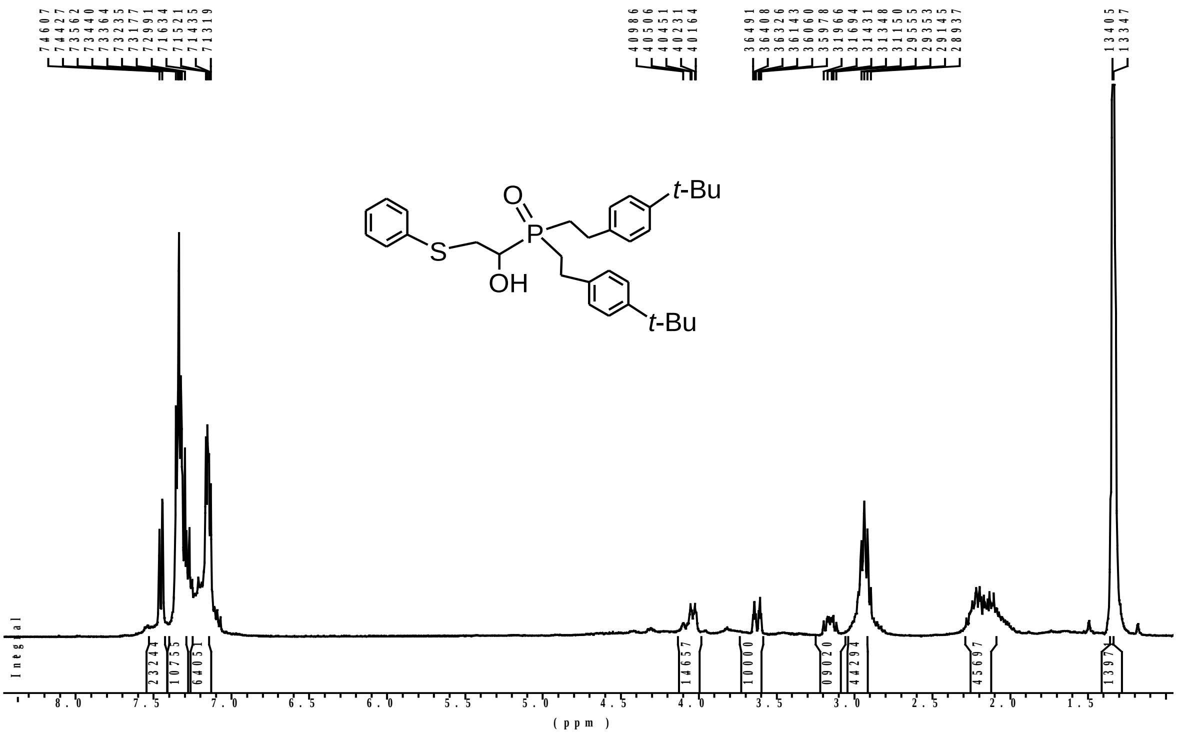

^{13}C NMR spectrum of phosphine oxide **3f** ($CDCl_3$)

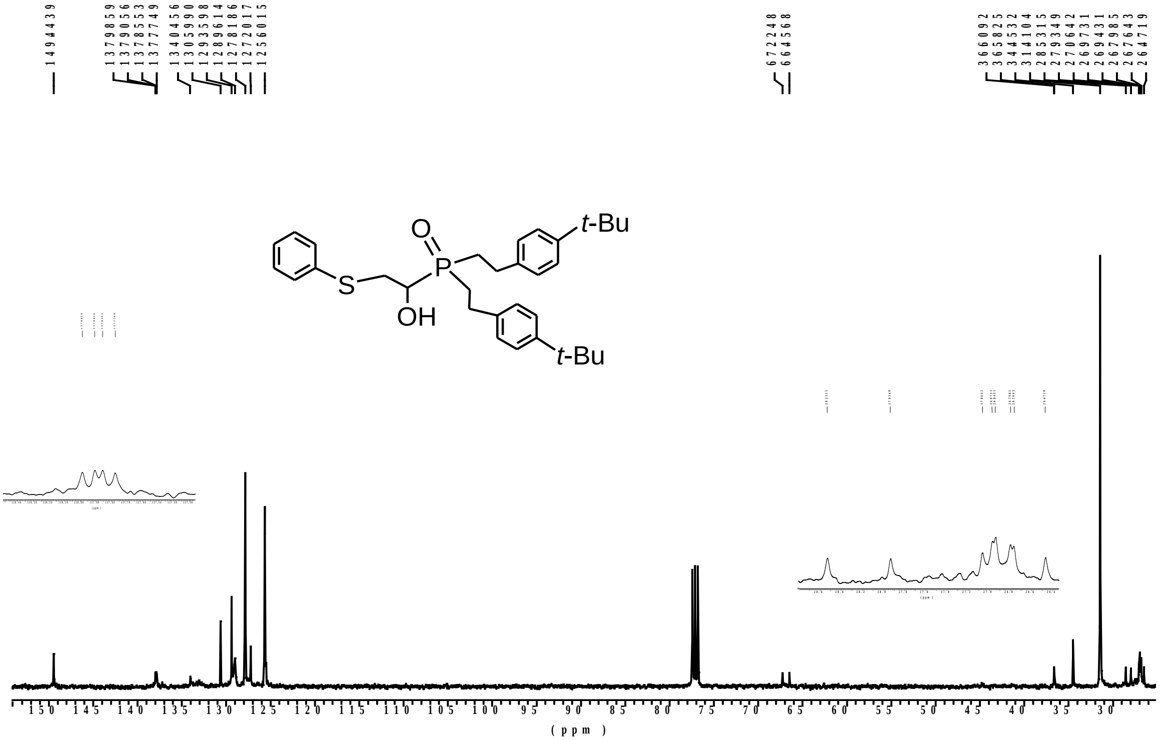

^{31}P NMR spectrum of phosphine oxide **3f** ($CDCl_3$)

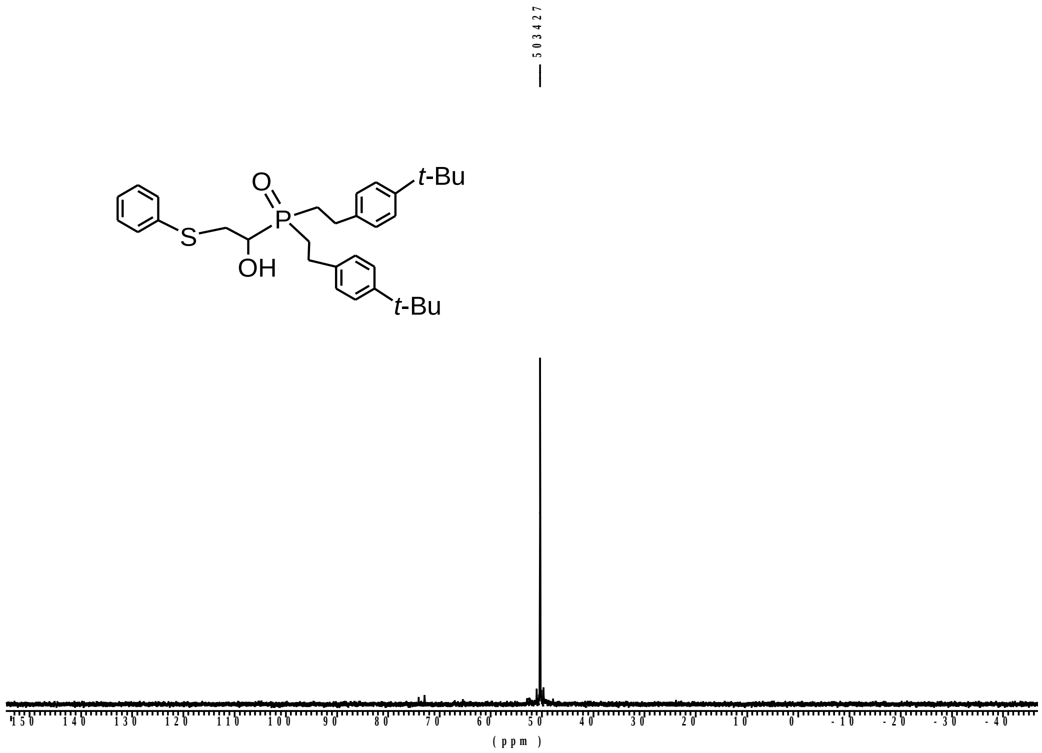

^{1}H NMR spectrum of phosphine oxide **3g** ($CDCl_3$)

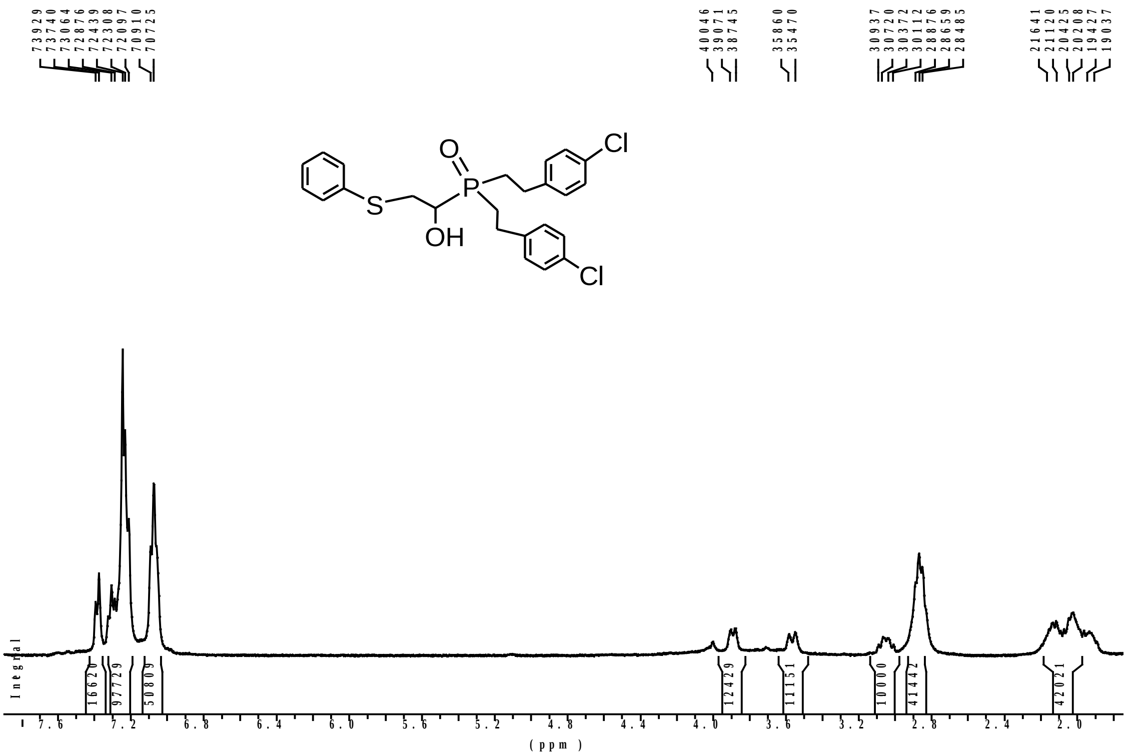

^{13}C NMR spectrum of phosphine oxide **3g** ($CDCl_3$)

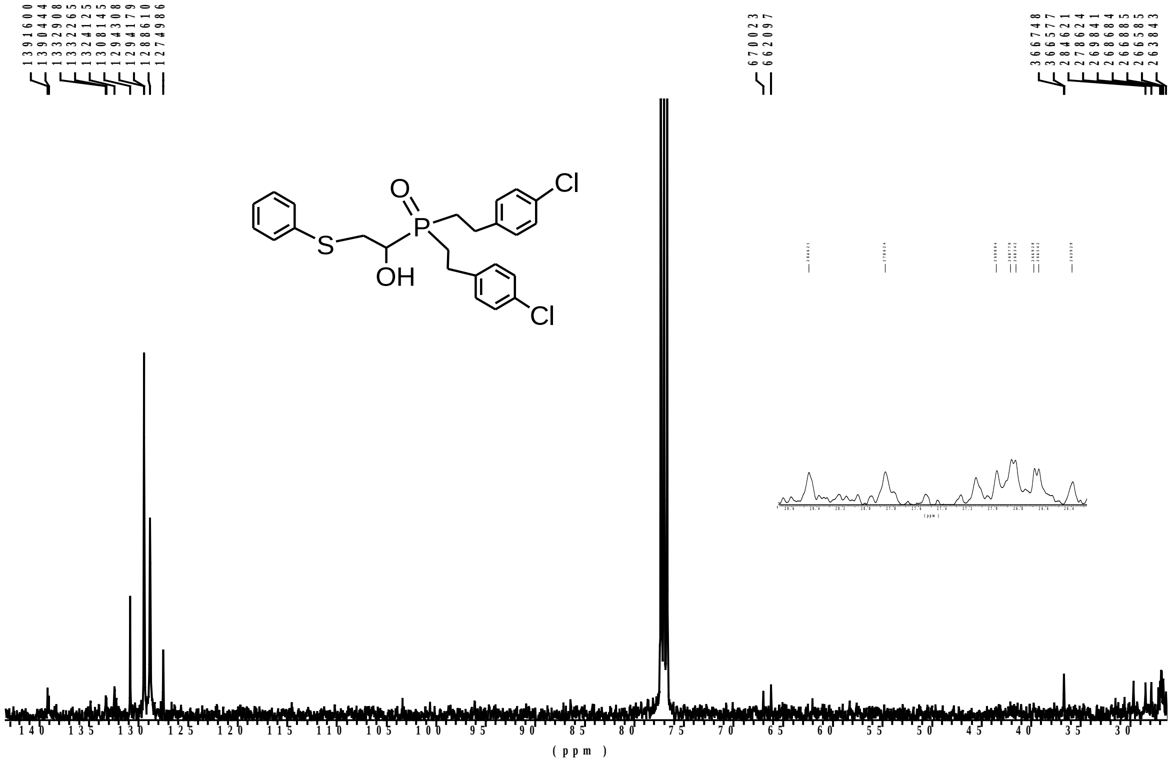

^{31}P NMR spectrum of phosphine oxide **3g** ($CDCl_3$)

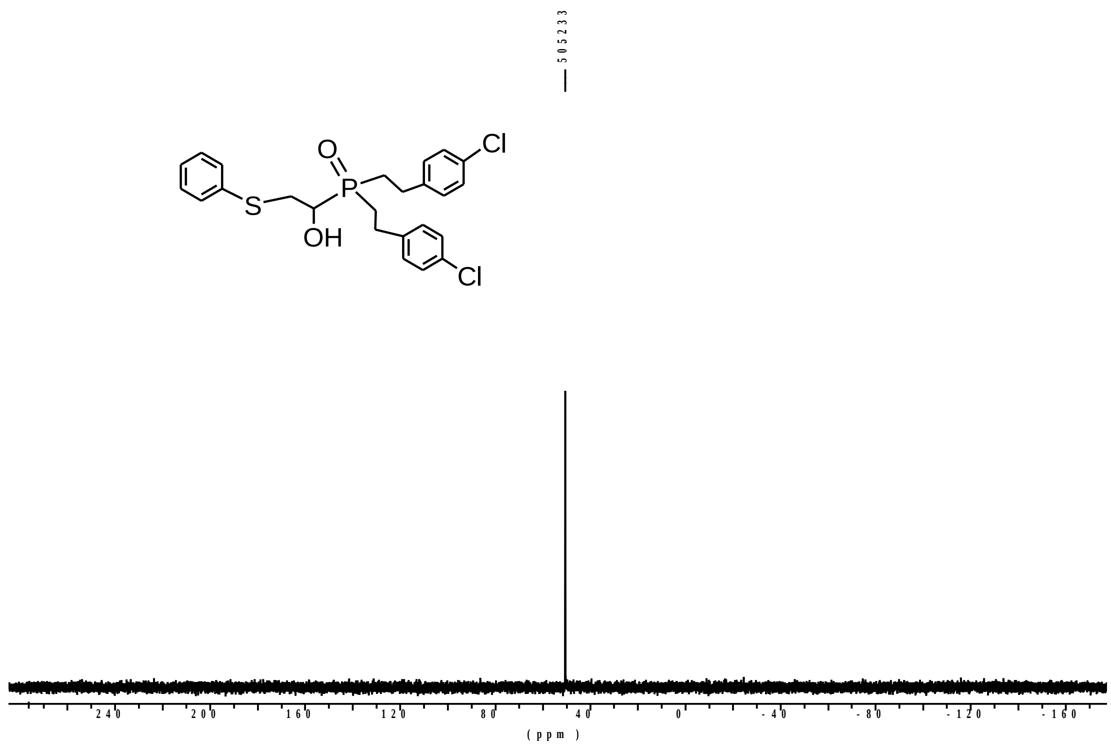

^{1}H NMR spectrum of phosphine oxide **3h** ($CDCl_3$)

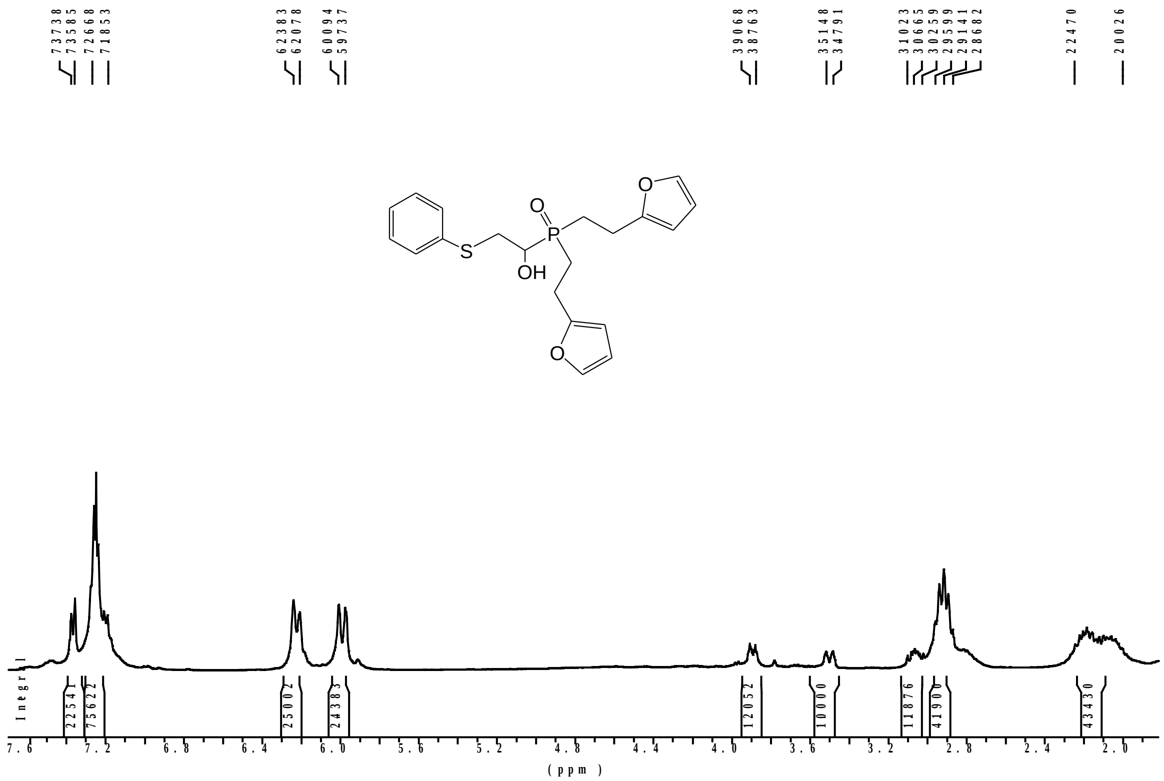

^{13}C NMR spectrum of phosphine oxide **3h** ($CDCl_3$)

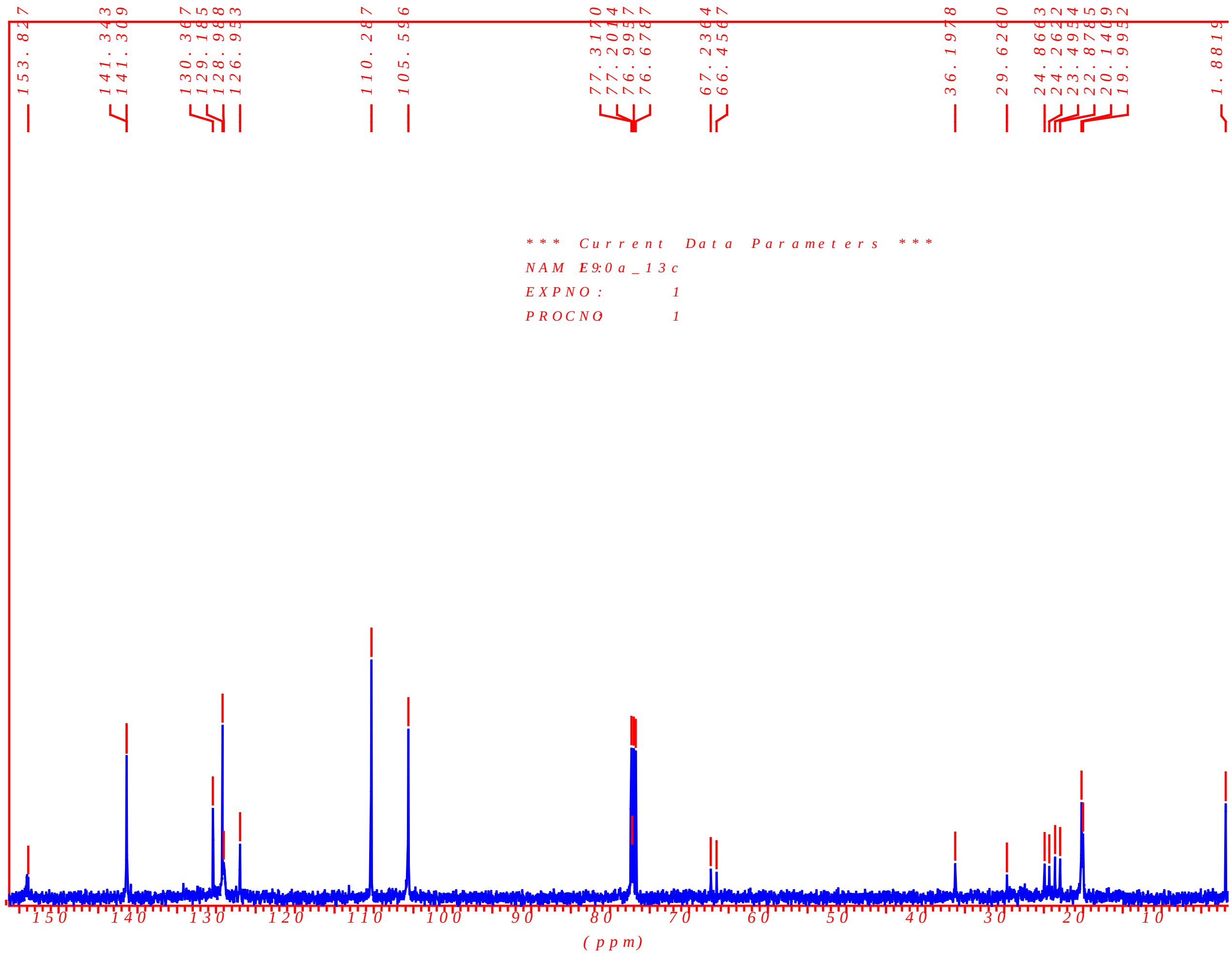

^{31}P NMR spectrum of phosphine oxide **3h** ($CDCl_3$)

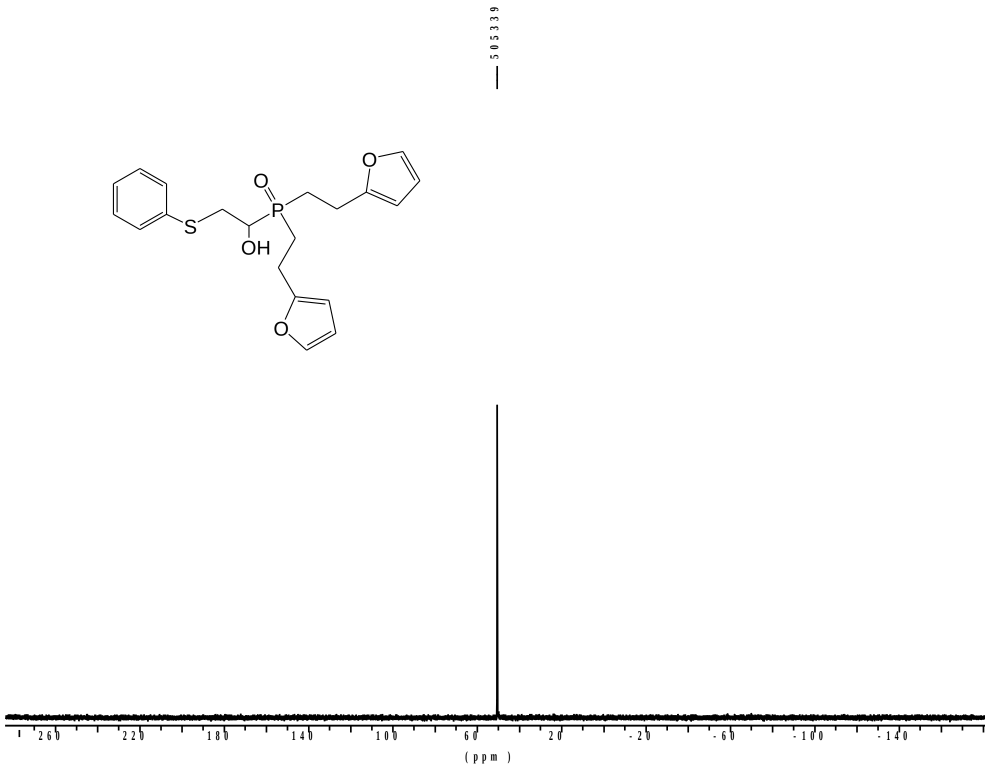

Supplement: File 1 — General remarks, experimental procedure and characterization data; crystallographic information for 3d; 1H, 13C & 31P NMR spectra of synthesized compounds. [file Beilstein_J_Org_Chem-11-1985-s001.pdf]
